# Antecedents of data analytics adoption: A systematic literature review from 2018-2024

**DOI:** 10.12688/f1000research.170252.1

**Published:** 2025-10-02

**Authors:** Alqa Husni, Wasanthi Madurapperuma, Ranpati Dewage Thilini Sumudu Kumari

**Affiliations:** 1 Business School, Informatics Institute of Technology, Colombo, Western Province, Sri Lanka; 2 Department of Accountancy, University of Kelaniya Faculty of Commerce and Management Studies, Kelaniya, Western Province, Sri Lanka; 3 Central bank of Sri Lanka, Colombo, Sri Lanka

**Keywords:** data analytics adoption, learning analytics, big data, systematic literature review, bibliometric analysis, theoretical framework

## Abstract

**Background:**

The rise of data analytics adoption has transformed multiple industries through technological advancements. However, utilizing big data analytics presents challenges that depend on adoption models used by individuals or organizations. Whilst numerous models on big data analytics exist, understanding the most influential theories shaping research in this domain remains limited. The study systematically explores the antecedents of data analytics adoption, aims to map the evolution of the field and uncover underexplored domains and integration gaps.

**Methods:**

A rigorous systematic literature review of 43 peer-reviewed articles published between 2018 and 2024, collected mostly from Scopus and Web of Science databases, was conducted, employing the Preferred Reporting Items for Systematic Reviews and Meta Analysis (PRISMA) guidelines and specific inclusion/exclusion criteria. Advanced bibliometric tools like VOSviewer and Microsoft Excel were employed to identify key trends, thematic clusters and integration gaps.

**Results:**

The study reveals research concentration in manufacturing sectors and developed Asian countries. The review identifies five interconnected adoption dimensions: technological; organizational; environmental; individual; and data-related factors. The Technology-Organization-Environment (TOE) framework dominates organizational-level studies, while the Unified Theory of Acceptance and Use of Technology (UTAUT) primarily guides individual-level investigations. Having identified five key research clusters, the review highlights that theoretical fragmentation persists between behavioral and resource-based perspectives.

**Conclusion:**

This study synthesizes the theoretical model of big data analytics research, providing guidance for future researchers in selecting an appropriate theoretical framework, differentiating between individual and organizational adoption levels and identifying significant determinants for technology adoption studies.

## 1. Introduction

Big data analytics (BDA) marks a pivotal milestone in many sectors for several purposes. While there has been an apparent surge on data analytics research recently, comprehensive systematic reviews remain limited. BDA is an advanced analytical technique of data management which helps to create meaningful insights that aid complex decision-making. ^1, 2^ Power et al. ^3^ claimed that business analytics and data analytics are specific subtypes of analytics where diagnostic, predictive and prescriptive subcategories rooted within the types. Policy makers and managers in contemporary business environments that are changing rapidly prefer to make decisions which are based on real-time data rather than relying on their internal insights. The implementation of BDA has emerged as a pivotal force influencing various sectors on a global scale, fundamentally altering organizational operations and decision-making processes. As of 2023, nearly 92% of universal digital leaders stated that their companies had adopted cloud technology to a certain extent. ^4^ Big data analytics was the second most popular adopted technology with around 61% adoption rate, and by 2027, global digital transformation spending is forecast to reach USD 3.9 trillion. ^4^ As firms increasingly recognize the importance of insights derived from data, the integration of BDA has become essential for maintaining competitive advantages and enhancing operational efficiency.

This trend is particularly evident in sectors such as healthcare, finance, retail, and education, where the ability to analyze large volumes of data can lead to improved outcomes and strategic advantages. ^5, 6^ Despite its potential, the global adoption of BDA has not been uniform. Most businesses responding to a 2023 survey conducted by Statista Research Department stated that investment in data and analytics was a top priority. However, only 37% mentioned that their efforts to improve data quality had been successful, highlighting an ongoing challenge faced by organizations across industry sectors. ^4^ Elgendy et al. ^7^ argued that there is a sheer need for data-driven cultures where data is treated as a significant asset at any organization. Various factors influence the rate and success of BDA implementation, including technological readiness, organizational culture, and external pressures. ^8– 10^ For instance, organizations with a robust technological infrastructure and a culture that embraces innovation are more likely to adopt BDA effectively. Conversely, those facing challenges such as data silos, lack of skilled personnel, or resistance to change may struggle to implement these technologies successfully. ^11, 12^ In the healthcare sector, BDA enhances patient care, streamlines operations, and improves research outcomes. By analyzing patient data, healthcare providers can identify trends, predict outcomes, and personalize treatment plans. ^6^ Similarly, in finance, BDA enables organizations to detect fraud, assess risks, and optimize investment strategies through real-time data analysis. ^13^ Retail establishments utilize BDA to gain insights into consumer behavior, optimize supply chain logistics, and enhance customer engagement. ^14^ By analyzing purchasing behaviors and preferences, businesses can tailor their products and marketing strategies to align more closely with consumer demands. Big data is a vital aspect of innovation, which has recently gained attention from academics and practitioners in the higher education sector. ^15^ In the realm of the education sector, using data analytics has augmented the capacity of institutions to monitor, evaluate and enhance student learning outcomes. By harnessing extensive datasets, universities are making informed decisions concerning student support, curriculum innovation and institutional management. This transformation has become evident within online education, particularly after the COVID-19 pandemic. As institutions transitioned to remote learning, data analytics emerged as a critical component in preserving academic continuity, providing novel methodologies to assess and evaluate students’ engagement and performance, the process defined as “learning analytics”. ^16^

In line with the practical side, studies on data analytics adoption have been growing in the past decade, coming from different fields or focuses. One of the first comprehensive bibliometric analyses on big data analytics adoption was published in 2021, spanning studies from 2014 to 2018. ^17^ The study employs 516 published papers to explore the trends, tools and techniques used in BDA adoption, particularly within the supply chain industry. In addition, this study conducted a Systematic Literature Review (SLR) of 79 papers summarizing the problem addressed in each paper and the proposed solution. This study provided a broad overview of big data analytics adoption, including applications, benefits and challenges across many sectors. The authors categorize the reviewed papers in this study into key areas, revealing that manufacturing and service industries are the most studied. Furthermore, the study highlights that the motivation for the research stemmed from the benefits of adopting BDA combined with a lack of sufficient research in the area.

The contributions of the present study to literature are three-fold. The study utilizes a differed approach in reviewing the literature in determining the factors of branches of data analytics adoption and adds to the novelty by linking the factors with the respective theoretical framework, thus enabling future researchers to develop new conceptual models that integrate two or more of the theories used in the past. The findings of this paper are expected to add to the literature of data analytics adoption and systematic review by offering an explicit topic related to data analytics adoption. However, this can also be extended to other forms of innovation and technology adoption, as the world is now evolving, and many organizations in different sectors give emerging technologies prominent priority. Another uniqueness of this paper is that it provides a holistic view of the antecedents of individual-level data analytics adoption. Employees play a pivotal role in the success of any organization. Therefore, employees need to adapt to novel technology and change for the organization to benefit.

The study seeks to address four main research objectives:

1. To explore publication trends, key authors and methodological approaches used in existing studies.
2. To map the thematic evolution and core focus areas in the BDA adoption literature from 2018 to 2024.
3. To identify the dominant theoretical frameworks and examine the extent to which individual, technological, organizational, and environmental factors influence BDA adoption outcomes.
4. To uncover underexplored domains, integration gaps, and future research opportunities within the BDA adoption-performance nexus.

This paper is organized as follows. Section 2 discusses similar research conducted in the area. Section 3 presents the Materials and Methods including the systematic literature review execution. Section 4 interprets the findings, followed by the discussion of results in Section 5. Finally, the conclusions in Section 6 summarizes the findings, considers the limitations, implications arising from the study and directions for future research.

## 2. Literature review

Studies on data analytics adoption have been growing over the past focusing on different domains across different sectors. However, limited literature reviews have been conducted, and the findings are often confined to a particular sector. One research closely related to the current study is a systematic review of the literature conducted by Ref. 18 to investigate the technological, organizational and environmental factors that affect the adoption of business analytics. The authors utilized the PRISMA technique to conduct an in-depth analysis of relevant research papers published between 2012 and 2022 ultimately selecting 29 articles for thorough examination. The researchers adopted the Technological-Organizational-Environmental (TOE) framework as an overarching theoretical lens to evaluate technology adoption at an organizational level. However, this study focuses solely on organizational adoption using the TOE framework. The authors highlight that future research could explore other theoretical frameworks and include a broader range of sources. Another systematic review conducted by Adrian et al. ^19^ using a relatively small sample of 18 relevant papers published between 2010 and 2017, identified ten key factors that influence the success of BDA implementation within organizations namely organization capability, human capability, analytics capability, analytics culture, environment, data management, data and information quality, system quality and perceived benefits. Aldossari et al. ^20^ also conducted a systematic literature review to identify key factors that influence big data analytics adoption in Small and Medium Enterprises (SME’s). The study aims to understand these factors to help SME’s effectively implement BDA and gain a competitive advantage, focusing on articles published between 2016 and 2022. After extracting 60 factors from the literature, a filtering process based on the frequency of citations narrowed it down to 21 factors, which were then sent to 10 private sector experts for ranking. The authors then identified 13 significant factors that are the highest influencers for BDA adoption in SMEs based on SLR expert ranking. However, the research has only been limited to SMEs and adoption at organizational level which emphasizes the need to explore other sectors. In another study, Al-Azzam et al. ^21^ highlights that although the knowledge in the area will expand due to the continuous enhancements in the development of big data application, academics are struggling to establish the key theories.

The existing literature, summarized in Table 1 has largely focused on determinants of BDA adoption, drawing from models such as the Technology Acceptance Model (TAM), Task-Technology Fit (TTF), and the Technology-Organization-Environment (TOE) framework. However, there is a noticeable gap in understanding how these adoption factors translate into firm-level value realization and strategic outcomes. Additionally, individual-level enablers, such as trust, user acceptance, and management support, are underexplored in their connection to organizational performance. The current research landscape also reveals theoretical and contextual fragmentation. For example, while Resource-Based View (RBV) and Dynamic Capabilities theories provide a strategic lens for understanding value creation, they are seldom integrated with adoption models. Moreover, specific application contexts such as logistics, public institutions, and learning analytics remain peripheral in empirical investigations.

**Table 1.** Previous systematic literature reviews and bibliometric analysis.

Author/s	Title of the study	Duration considered for the review	Number of articles in the sample	Findings of the study
Adrian et al. (2017)	Factors influencing the Implementation Success of Big Data Analytics: A Systematic Literature review	2010-2017	18 articles	Established 10 influencing factors that may influence the success of BDA implementation
Inamdar et al. (2021)	A systematic literature review with bibliometric analysis of BDA	2014-2018	516 articles For bibliometric analysis and 79 articles for systematic literature review.	Derived a new categorization of BDA of seven main areas in the supply chain and its applications in various sectors
Horani et al. (2023)	Determining the factors influencing Business Analytics adoption at Organizational level	2012-2022	29 articles	Investigates the technological, organizational and environmental factors that affect the organizational adoption of Business Analytics
Aldossari, Mokhtar, & Ghani (2023)	Factors influencing the adoption of Big Data Analytics: A Systematic Literature and Experts Review	2016-2022	60 articles	The study extracted 13 significant factors that are vital influencers for BDA in SMEs

Source: Authors Developed, 2025.

Due to these limitations, there is a rising need for a more structured and comprehensive review of data analytics adoption models and theories that can guide rigorous research in various settings, including diverse industries and economies, thus emphasizing the urgency in implementing data analytics to cater to the benefits it offers.

Given the rapid evolution of BDA research from 2020 to 2024, there is a timely need to systematically synthesize existing knowledge, map out emerging trends, and identify critical gaps to address this need. This paper undertakes a Systematic Literature Review (SLR) by analyzing peer-reviewed studies using bibliometric mapping and qualitative synthesis techniques.

## 3. Materials and methods

The literature has adopted diverse methodologies in conducting research on data analytics adoption. One such approach is a Systematic literature review (SLR). SLR represent a methodological approach to synthesizing scientific evidence aimed at addressing a specific research question in a manner that is reproducible, while ensuring that all published evidence pertaining to the subject matter has been included. ^18^ SLR is considered the appropriate methodological tool for the present study due to its ability to thoroughly summarize the influential determinants of data analytics adoption at both individual and organizational levels across different sectors. SLR follows a stepwise approach that first defines the aim of the review, formulates research questions, selects suitable evidence, evaluates the quality of evidence, collects data and analyze the results. ^20^

In this study, the systematic review adhered to the new PRISMA guidelines proposed by Page et al. ^22^ to ensure transparency and accuracy in reporting the findings. The SLR was conducted in three distinct stages as applied by Horani et al. ^18^ These stages include: (1) planning stage; (2) execution stage; and (3) summarizing stage as discussed in detail below.

### 3.1 Planning stage

The initial phase in the planning stage involves determining the need to conduct a systematic review. This arises from the attempt to address the research objectives listed in Section 1. Prior research has demonstrated that various factors are linked to the adoption or intention to adopt data analytics. Different dimensions envelop these factors. Nevertheless, to our knowledge, a limited number of literature reviews have systematically synthesized and differentiated which factors influence firm-level and individual-level adoption. Hence, the next phase was to define the strategies for article selection. During this phase, the researcher established selection criteria for the eligibility review, which are presented in Table 2. The inclusion/exclusion criteria ensured that the literature was relevant to the research objectives. To ensure that the included studies were relevant, the search was limited to factors influencing data analytics adoption across all sectors at different levels, employing different theories, as the main aim was to establish a linkage between the factors, adoption level and theory.

**Table 2.** Inclusion-Exclusion criteria.

Criteria	Inclusion	Exclusion	Rationale
Type of publication	Journal articles	Other types of publications, such as conference papers, books, and dissertations	To ensure that publications met the standards of academic rigor and had undergone peer review
Type of Study	Empirical studies, case studies	Systematic literature reviews, meta-analysis, bibliometric analysis, content analysis, conceptual frameworks without testing	To ensure the search is focused on answering the addressed research questions
Publication year	2018-2024	Publications prior to 2018 and after 2024	To ensure that the literature is relevant and up to date for forecasting trends in innovation
Language	English	Non-English	English is the official language in publishing

Source: Authors Developed, 2025.

### 3.2 Execution stage

**Figure 1.**
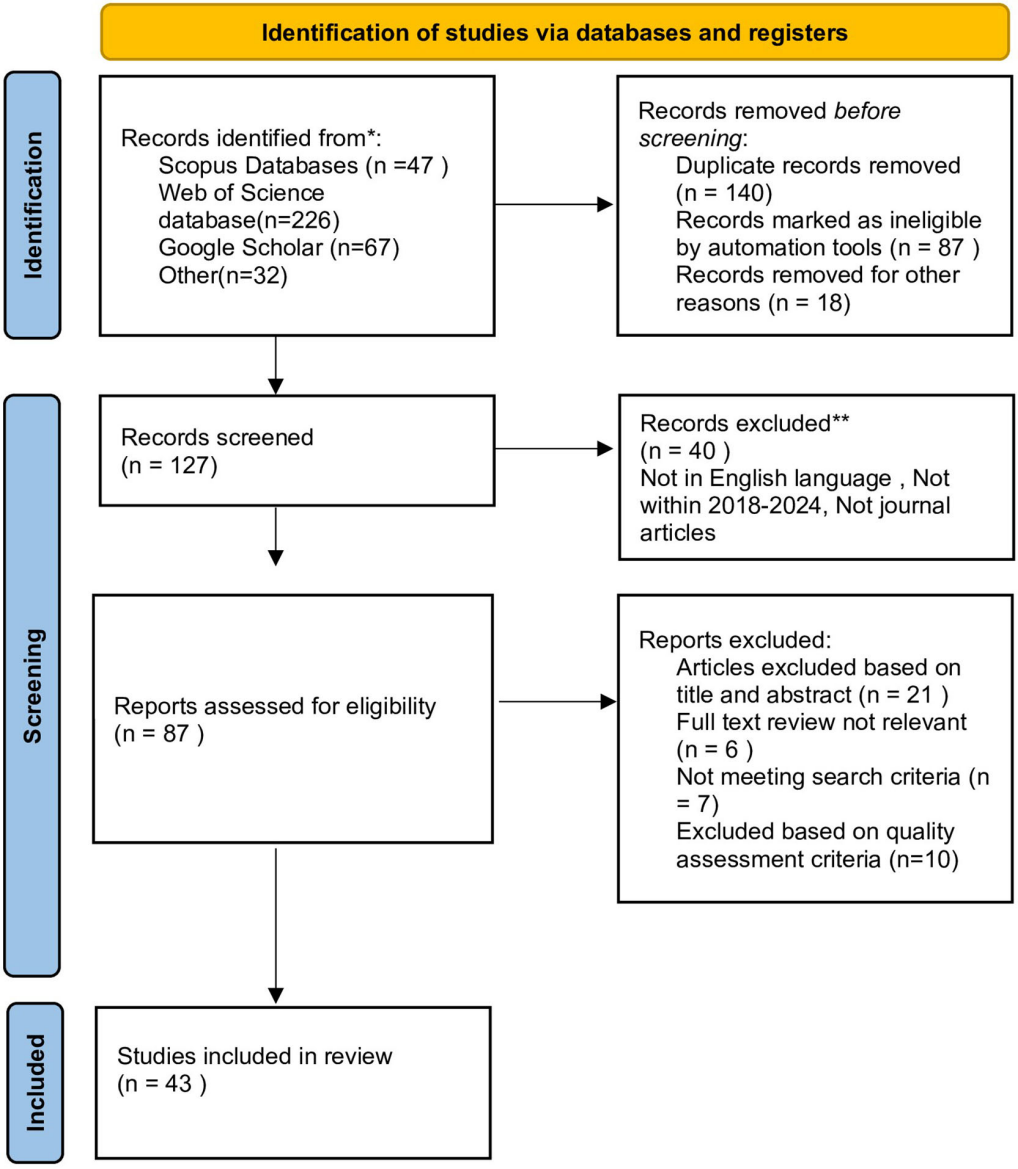
Search results. Source: Authors’ Findings, 2025. Alt Text: PRISMA Flow diagram explaining the steps for each filtering stage.

This study mainly uses the Web of Science and Scopus databases as they are the most trusted and reputable indexing bodies, consisting of articles published in peer-reviewed journals. The PRISMA model was used to select the sample size for analysis. PRISMA is a well-established evidence-based reporting mechanism used in systematic reviews. ^18^ The data collection process for this review follows the PRISMA flow diagram. The review was conducted between January 2025 and March 2025. Four main steps were followed in this stage. As the first step, a literature search was conducted using the following search strings: “data analytics” OR “big data analytics” OR “business analytics” OR “learning analytics” AND “adoption” OR “intention” AND “factors” OR “determinants” OR “antecedents”. The search and the screening of the documents were conducted in several stages. To obtain the relevant literature on the factors determining data analytics adoption in the education sector, firstly a broad search was done across all sectors. As the second step, using the screening based on the above keywords, publication period: 2018-2024, type: journal articles, language: English, a sample was selected. As the third step, only documents related to the study were screened and articles were removed if the abstract was irrelevant to the search. The articles were subjected to a manual review process. As the final step, all refined articles were thoroughly reviewed based on the full text and only articles relevant to the research questions were chosen for the study. Figure 1 shows the PRISMA model being utilized.

### 3.3 Summarizing stage

The initial search process, utilizing keywords stated in the previous stage, resulted in a total of 372 articles. After the removal of duplicates, 127 articles were retrieved. Filters were then applied. This process led to a set of 87 articles to be considered. Following this, the researcher performed a manual review by skimming the titles and abstracts to determine the relevance of the retrieved articles, with a specific focus on empirical articles that are closely associated with the topic of the study. The remaining articles were screened further by reviewing the full-text content, which led to 43 articles being classified as relevant to the topic of this study, and 44 were identified as irrelevant and discarded. The study was systematically reviewed with bibliometric analysis conducted using VOS viewer software and Microsoft Excel.

## 4. Results

This section reports the main findings from the systematic literature review with the aim of addressing the research objectives listed in Section 1. This section is structured into three subsections. While Section 4.1 conducts a descriptive analysis of the selected studies, Section 4.2 identifies key theoretical frameworks and determinants, and Section 4.3 outlines results from the keyword, content and co-authorship analysis.

### 4.1 Descriptive analysis

This section reports findings of the review through the distribution of studies by year, key journals, citations, contributing authors, sectors, level of analysis, geographical region and research approach.

4.1.1 Chronological distribution of chosen studies (Publication trend)

**Figure 2.**
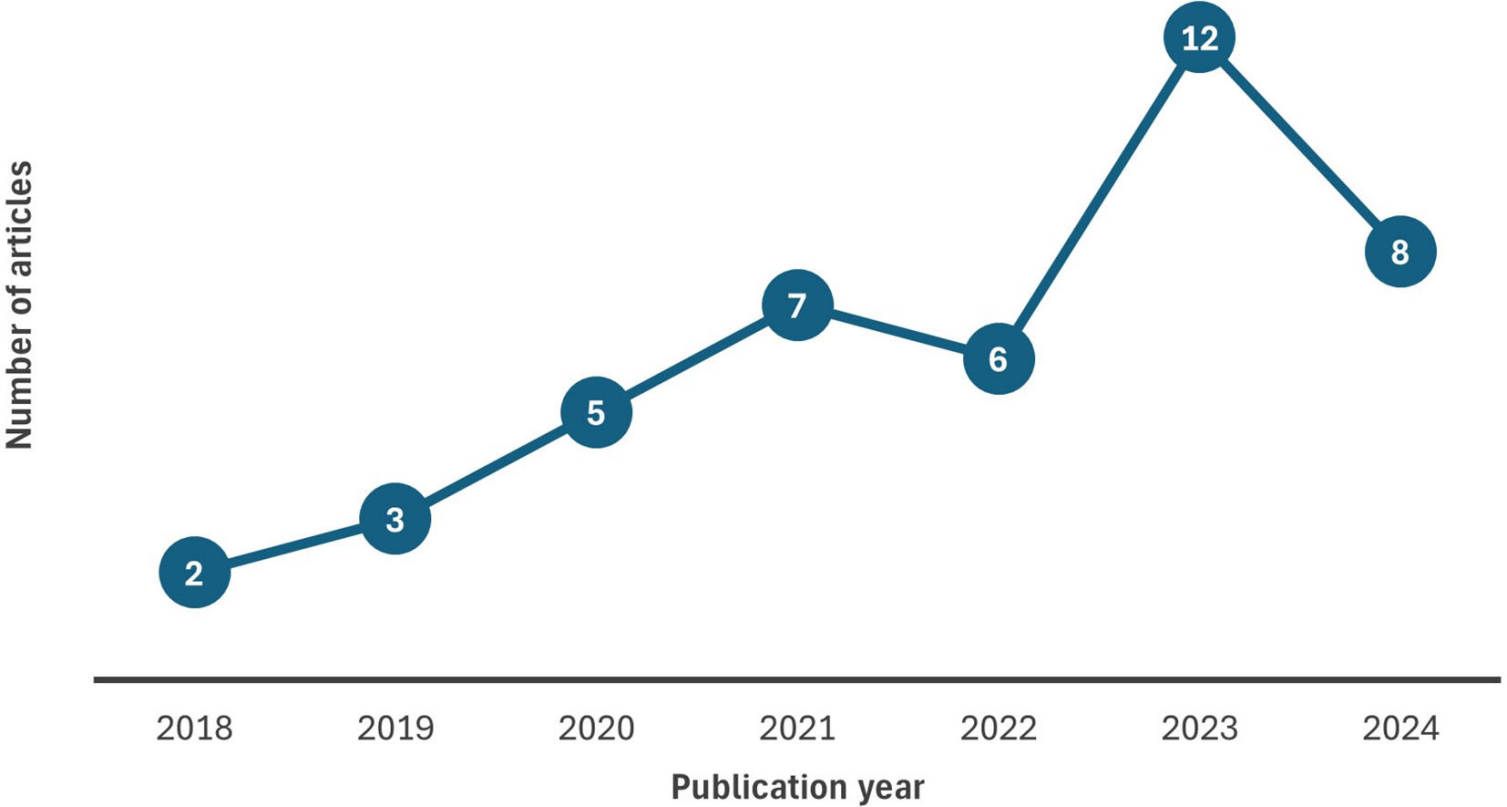
Chronological distribution of chosen studies. Source: Authors’ Findings, 2025. Alt Text: A line graph representing the number of articles published over the period 2018-2024.

Figure 2 illustrates the publication trend of selected articles considered in this review spanning 2018 to 2024. The results indicate that research on big data analytics adoption is gradually increasing over time with most documents published in 2023. The number of articles in the year 2024 will undoubtedly be higher than shown in the figure due to the time lag in publications. Further, this growing trend highlights the importance of adopting data analytics and its related technologies indicating the priority that various domains place data analytics adoption for their decision making. It is noteworthy that eight studies have been published in 2024, however, it is too early to forecast that attention to BDA may decline with time

4.1.2 Distribution of Journals

The number of publications on specific topics represents an important indicator for authors. The number of selected papers for our study ^23^ is an essential indicator of the potential for exploring research topics. The articles considered for review were from at least 34 different journals. The analysis results are in Table 3. Sustainability was the journal with the highest number of publications. ^5^ Information Systems Frontiers, Management Decision, Construction Economics and Building. Resource Mang, J and Journal of Business Analytics are other journals that have published at least two articles on this research topic, confirming them as the most cited journals so far. International Journal of Management, International Journal of Logistics Management, Sustainability, Industrial Marketing Management, and Journal of Big Data are notably the journals publishing articles with the most citations thus far, as depicted by Table 4.

**Table 3.** Publications per Journal.

Journal Title	Count of Publication Title
Sustainability	5
Information systems frontiers	2
Management decision	2
Construction Economics and Building	2
Inf. Resour. Manag. J.	2
Journal of business analytics	2

Source: Authors’ Findings, 2025.

**Table 4.** Citations per Journal.

Journal title	Sum of citations
International journal of information management	486
The international journal of logistics management	357
Sustainability	301
Industrial marketing management	227
Journal of big data	221
Ind. Manag. Data syst.	208
Journal of retailing and consumer services	165
Journal of computer information systems	128
Journal of open innovation: technology, market, and complexity	98
Inf. Resour. Manag. J.	95
Management decision	68
Decision science letters	48
Construction economics and building	46
Journal of decision systems	34
Australasian journal of educational technology	29
Enterprise information systems	27
Journal of business analytics	25
Managerial auditing journal	23
Technological forecasting and social change	22

Source: Authors’ Findings, 2025.

4.1.3 Citations and most influential authors

**Figure 3.**
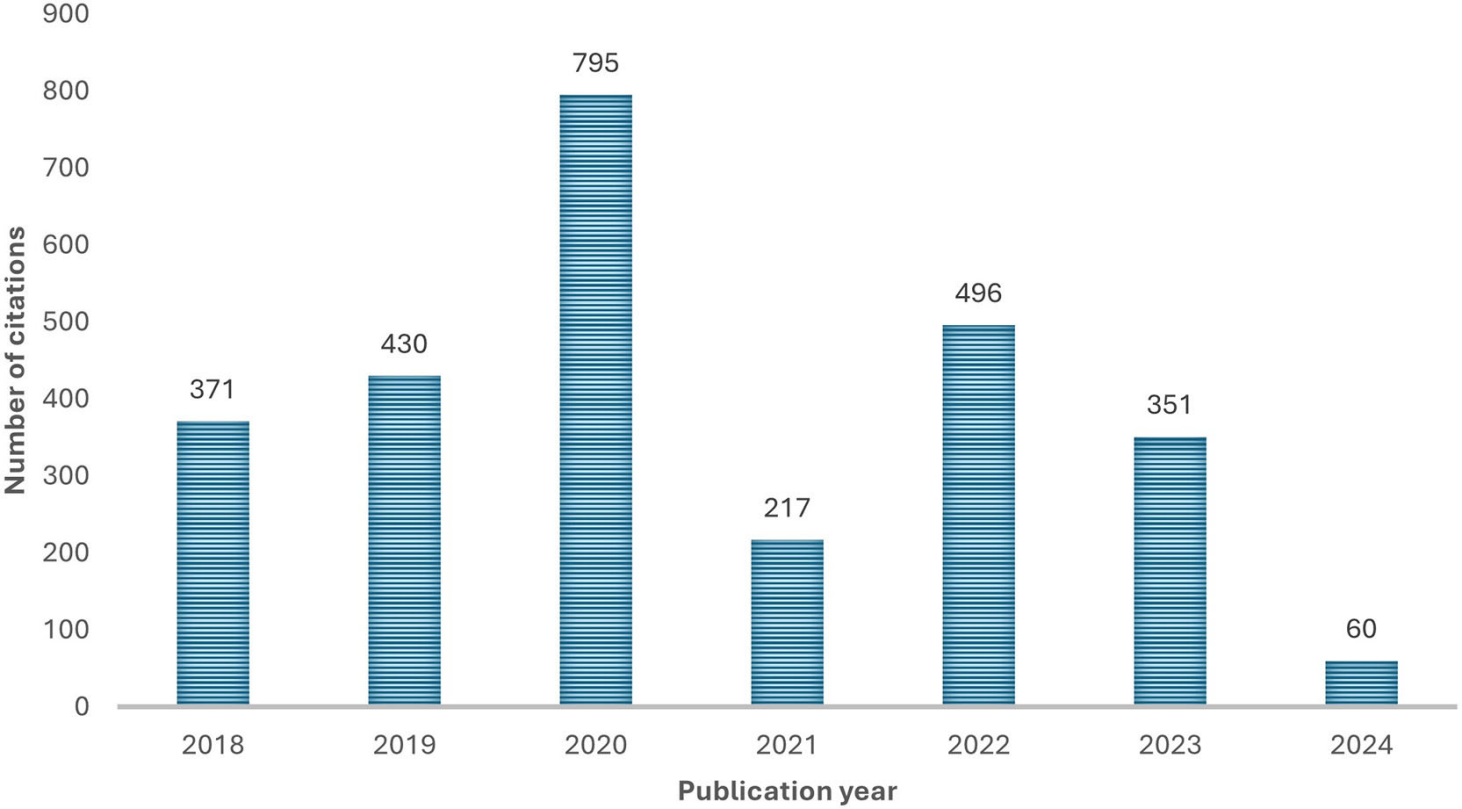
Number of citations per year. Source: Authors’ Findings, 2025. Alt Text: A bar graph representing the total number of citations each year from 2018-2024.

Figure 3 shows the evolution of citations by years during the period 2018-2024. The number of citations increased between 2018 and 2020, 2020 marking the highest number of citations. There is a notable drop in 2021 and a decrease in the number of citations from 2022 to 2024. Despite the limited portion of 2024, analysis suggests that there is limited interest for the papers published in 2024.

As part of the bibliometric analysis, key authors in this field are considered as the authors with the highest number of citations. The influential authors whose work has been cited at least 19 times are represented in Table 5. Namely, Maroufkhani, Parisa; Lutfi, Abdalwali; Lai, Y and Sun, Shiwei are a few of the most cited authors in this domain. These authors have considered single sector organizational adoption of big data analytics.

**Table 5.** Influential Authors.

Authors	Total number of citations
Maroufkhani, Parisa	694
Lutfi, Abdalwali	410
Lai, Y.	357
Sun, Shiwei	227
Shahbaz, M	221
Park, Jong-Hyun	128
Sekli, Giulio Franz Marchena	98
Verma, Surabhi	81
Baig, MI	59
Al-Azzam, MKA	48
Iranmanesh, M	45
Alaskar, T.	34
Clark, JA	29
Sharma, Mahak	27
Aghimien, DO	27
Islam, S	23
Shafique, Muhammad Noman	22
Chaurasia, Sushil S.	19

Source: Authors Developed, 2025.

4.1.4 Distribution of chosen studies by sector

**Figure 4.**
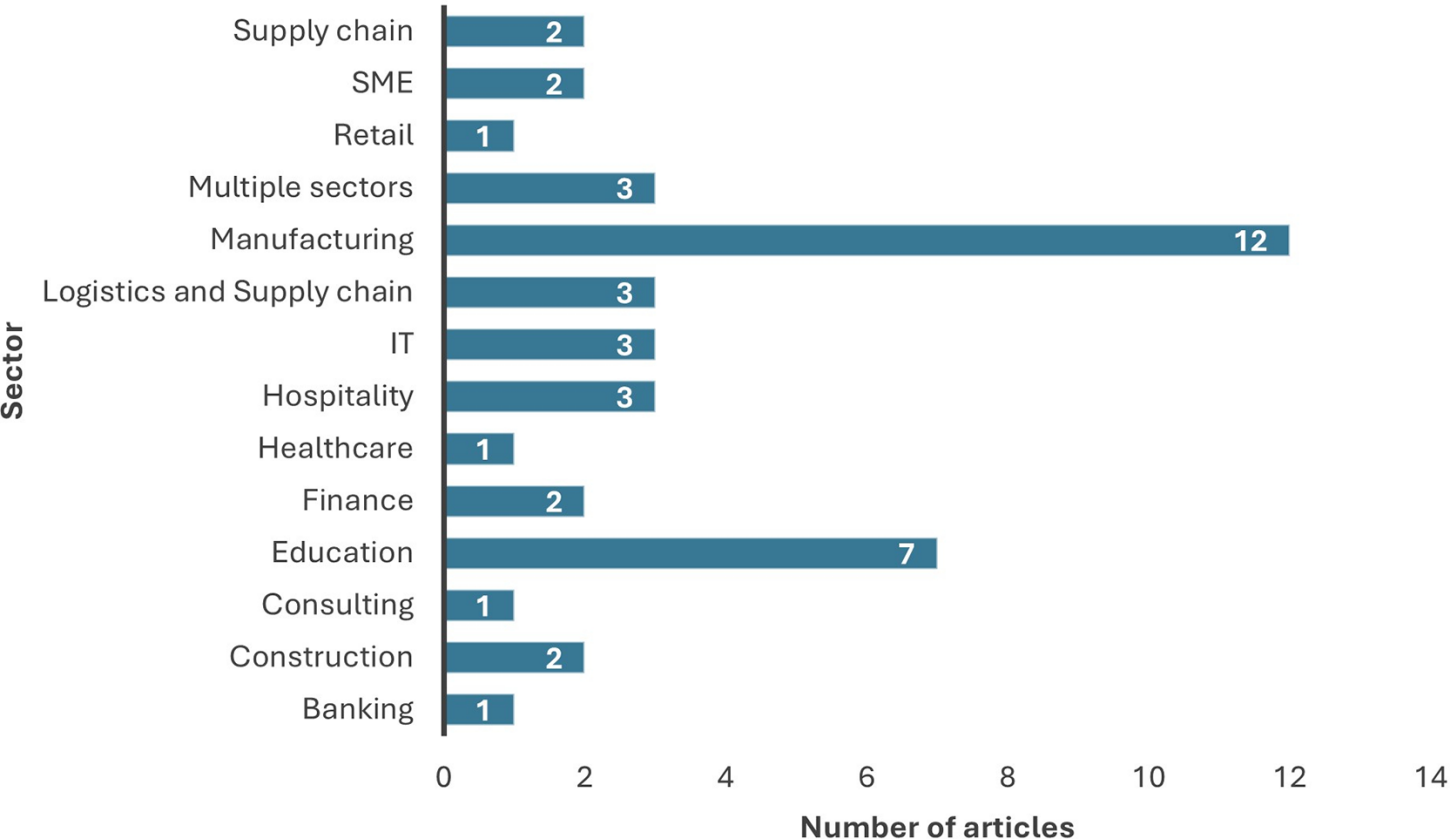
Distribution of articles sector wise. Source: Authors Developed, 2025. Alt Text: A bar graph representing the number of publications conducted for each sector.

**Figure 5.**
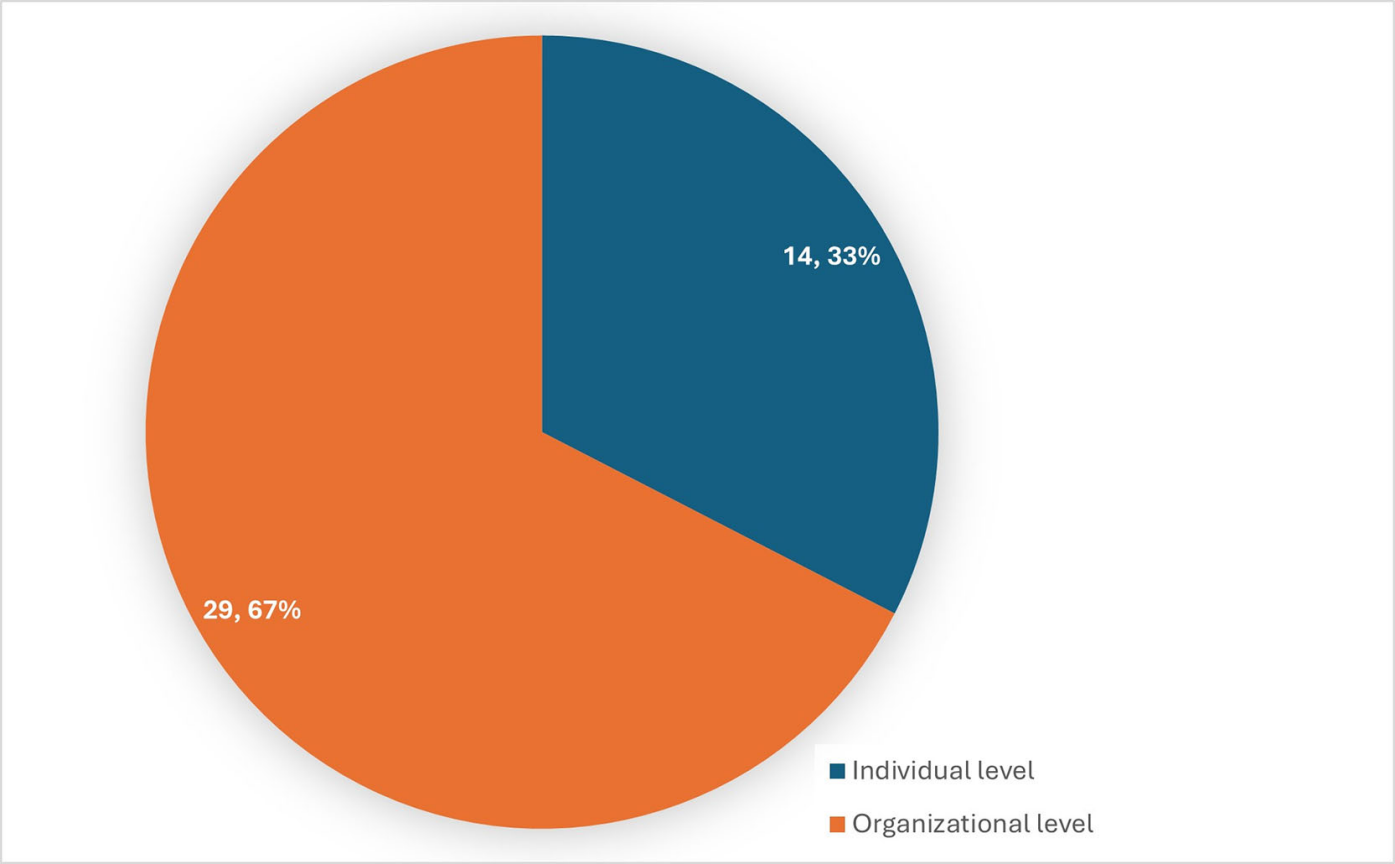
Distribution of the level of analysis. Source: Authors Developed, 2025. Alt Text: A pie chart representing the distribution of articles considering individual and organizational level analysis.

From the review of the selected 43 papers, the manufacturing sector emerged as the most prominent research conducted for data analytics adoption, as apparent from Figure 4. Researchers have also researched the education sector; however, most of the studies focused on the organizational level of adoption and its impact on organizational performance. Nevertheless, the employees of the organization need to prioritize the adoption initially for the organization to benefit from the usage. This emphasizes the need for future research to conduct individual level analysis prior to considering the level of adoption at organizational level. Most of the studies have been conducted at the organizational level and explored the impact of organizational adoption of data analytics on organizational measures. Figure 5 shows the distribution of level of analysis in the studies considered for review.

4.1.5 Distribution of chosen studies by geographical region

**Figure 6.**
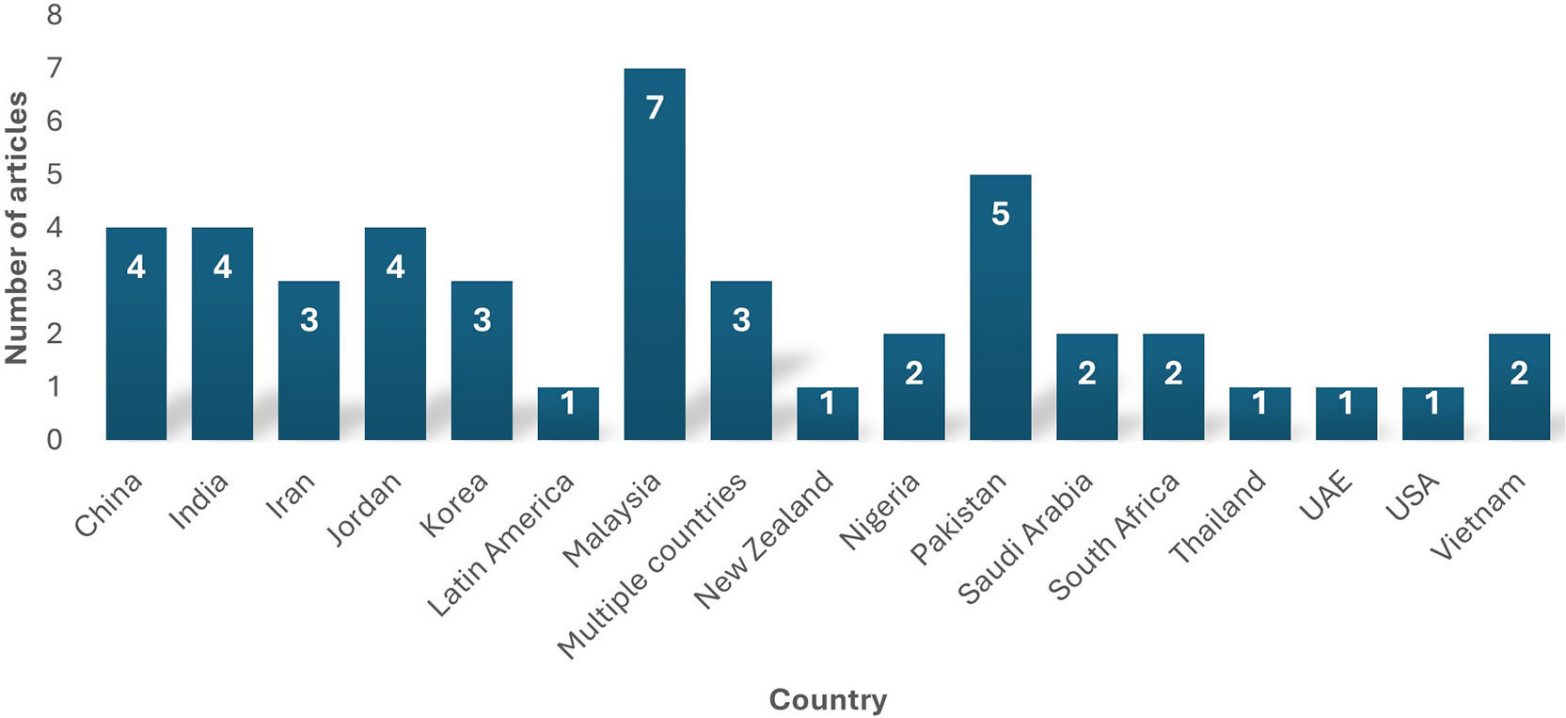
Geographical distribution of articles. Source: Authors Developed, 2025. Alt Text: A bar graph representing the number of studies conducted on each country.

The 43 selected studies for this review span at least 17 countries, as depicted by Figure 6. Malaysia contributed to most studies in data analytics adoption. In summary, most of the data analytics adoption research for this review was carried out in developed countries, thus highlighting the need for future researchers to focus on developing countries and identify the status and barriers of data analytics adoption amongst them. The study also indicates that most Asian countries have been involved in data analytics research, where it has a notable presence.

4.1.6 Distribution of chosen studies by research approaches

**Figure 7.**
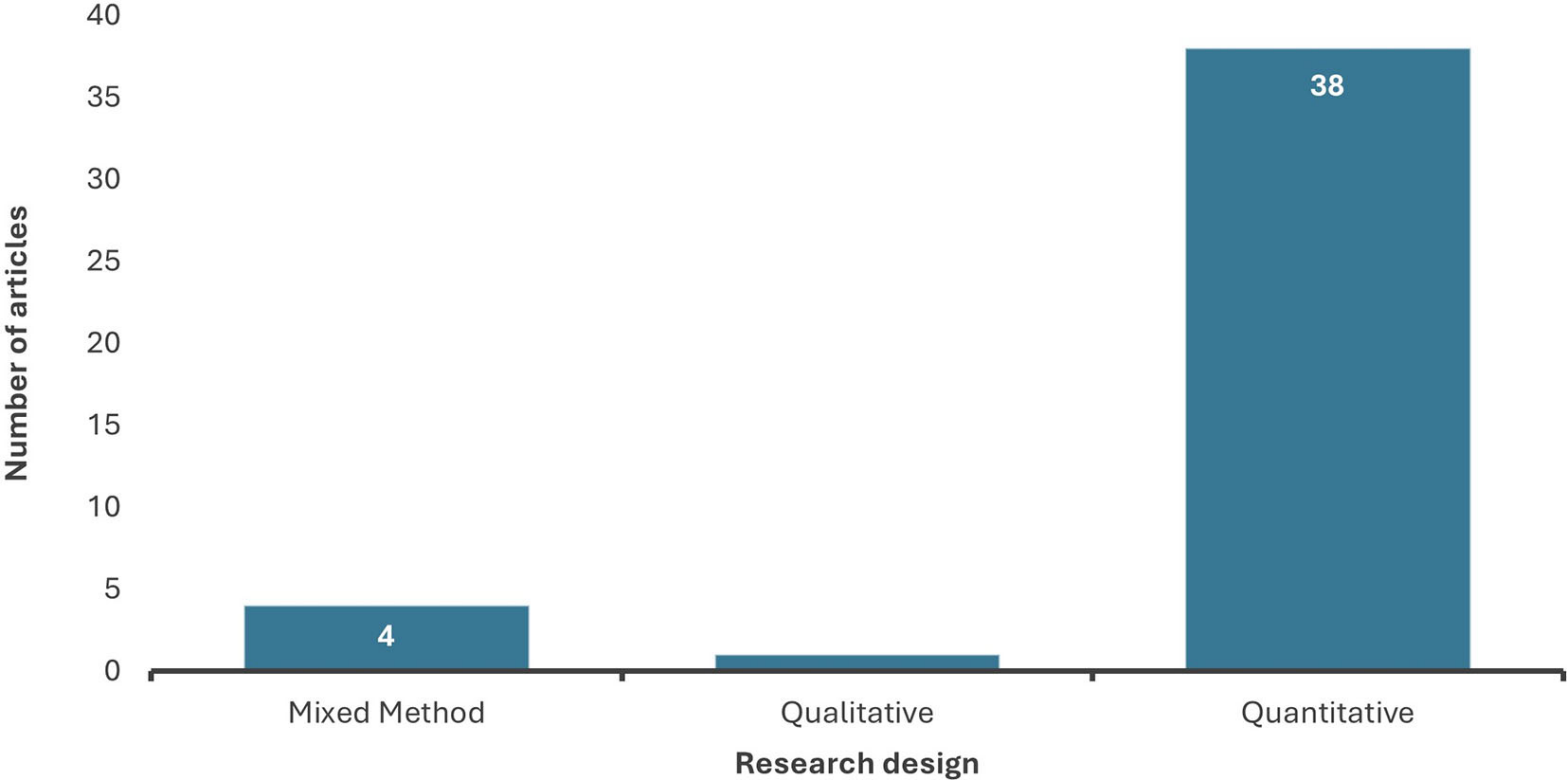
Distribution of research design. Source: Authors Developed, 2025. Alt Text: A bar graph representing the distribution of methodological approaches used in past studies.

The analysis revealed that most of the selected studies employed the quantitative research approach. In comparison, qualitative research was utilized only in one study, while mixed methods constituted 4 of the chosen articles, as presented in Figure 7. These statistics indicate that data analytics research is based on strong empirical evidence to quantify the relationship between the variables and the level of data analytics adoption, further evaluating its impact on the performance of the firm or individual.

### 4.2 Landscape of theories and determinants

This section systematically analyses the key determinants and respective theoretical frameworks.

4.2.1 Influential theoretical frameworks

The SLR analysis demonstrates that the TOE (Technology Organization Environment) was the top dominating theory heavily used to identify factors influencing organizational adoption of data analytics. The TOE framework developed by Ref. 24 is used to examine how technological, organizational and environmental contexts affect organizational performance. ^25^ It is a well-established framework for understanding technology adoption and has been applied to various forms of innovation. As observed, TOE has been coupled with TAM (Technology Acceptance Model) ^26^ and DOI (Diffusion of Innovation) by a few researchers. ^1, 27, 28^

The second most employed theoretical model is the UTAUT (Unified Theory of Acceptance and Use of Technology). Developed by Venkatesh et al., ^29^ UTAUT is aimed at providing an insight into all factors which influence the behavioral intention towards the use of a new technology. Widely used for the adoption of new technology from an individual perspective, UTAUT uses the constructs performance expectancy, effort expectancy, social influence and facilitating conditions as determinants of technology adoption. ^29^ However, the results of our review reported contrasting conclusions for each construct in different sectors. This highlights the need to employ UTAUT in the context of future research.

The Technology Acceptance Model (TAM), developed by Ref. 30, provides a robust framework for exploring how users accept new technologies, focusing on perceived usefulness and perceived ease of use. A few researchers ^12, 21, 26, 31^ have employed TAM as research has consistently shown that PU and PEOU are reliable predictors of users’ behavioral intentions and technology use. However, studies also demonstrate that the TAM does not account for human capabilities and practical knowledge, despite describing an individual’s motivations for using the system. ^21^ Future research can consider integrating TAM with other adoption models that do consider human capabilities and practical knowledge constructs. Empirical investigations have indicated that adoption of emergent technologies may necessitate the incorporation of “soft skills” alongside behavioral intentions, technical proficiencies and domain-specific knowledge. ^21^ The study further adds that it is imperative to account for social influences, belief systems and contextual factors when advocating for the adoption of novel technologies. According to Olufemi ^32^ the TAM overlooks business requirements, such as the cost of technology, which have a significant effect on the capacity to adopt particular technologies. In addition, the study observed that the TAM did not consider crucial acceptance requirements for major organizational technologies, such as the support of top management, the perception of privacy and security, and organizational culture. Thus, there are a variety of different factors that need to be considered when implementing novel technology at any organization which may require the integration of multiple theoretical frameworks.

Diffusion of innovation theory (DOI), developed by M. Rogers in 1962 has been widely used to explain the innovation diffusion process. The theory explains how new ideas and technologies spread through a population over time. According to Ref. 33, the five steps in the innovation-decision process developed by Rogers are knowledge, persuasion, decision, implementation, and confirmation. Before decision makers make the decision to adopt or reject innovation, they first need to comprehend the innovation, identify the potential benefits of adopting it, and then develop an attitude towards it. The process that technology diffuses is thus not solely linked to its distinctive capability to address technical challenges, but is also intertwined with the internal organizational framework, external organizational attributes, and leaders’ attitudes toward transformation. Both Innovation and organizational characteristics play a significant role in the assimilation of a novel technology. ^9^

Task technology fit and Institutional Theory are other theories that have been gaining attention in the recent past.

Institutional Theory is a theoretical framework in organizational studies that examines how organizations are influenced by their social and cultural environments. It focuses on how rules, norms, and routines become established as authoritative guidelines for organizational behavior. Despite the attributes of the technology itself, successful diffusion is dependent on the institutional willingness. The theory emphasizes the significance of regulative, normative and cultural cognitive components in influencing organizational decision-making processes. Hence, institutional theory is well-suited for explaining organizational behavior. ^9, 10^ Institutional theory has often been coupled with the TOE framework to provide a more comprehensive analysis of the environmental influences in BDA adoption. Future researchers can consider integration with other theories like UTAUT to enrich the theoretical underpinnings of the research.

Task Technology Fit (TTF) implies that the interplay between task characteristics and technology functionalities influences the effective adoption of a technology. ^34^ Task technology fit explains that the technology must be utilized and must be a good fit with the task it supports for it to have an impact on individual performance. ^35^ This study defines data quality, data location, access authorizations, data compatibility, ease of use/training, timeless manufacturing, system reliability, and user information system relationships as typical dimensions when measuring fit. Task technology fit has been employed for the analysis of the adoption of technology by individuals by several studies. ^34, 36^ It has been integrated with UTAUT and TAM, respectively.

TTF is consistent with the model proposed by Ref. 37 in that implementation and attitudes towards technology lead to individual performance impacts. Task technology fit is a critical construct providing a strong theoretical basis for understanding the impact of user involvement on performance, which was not enveloped by the earlier model.

**Figure 8.**
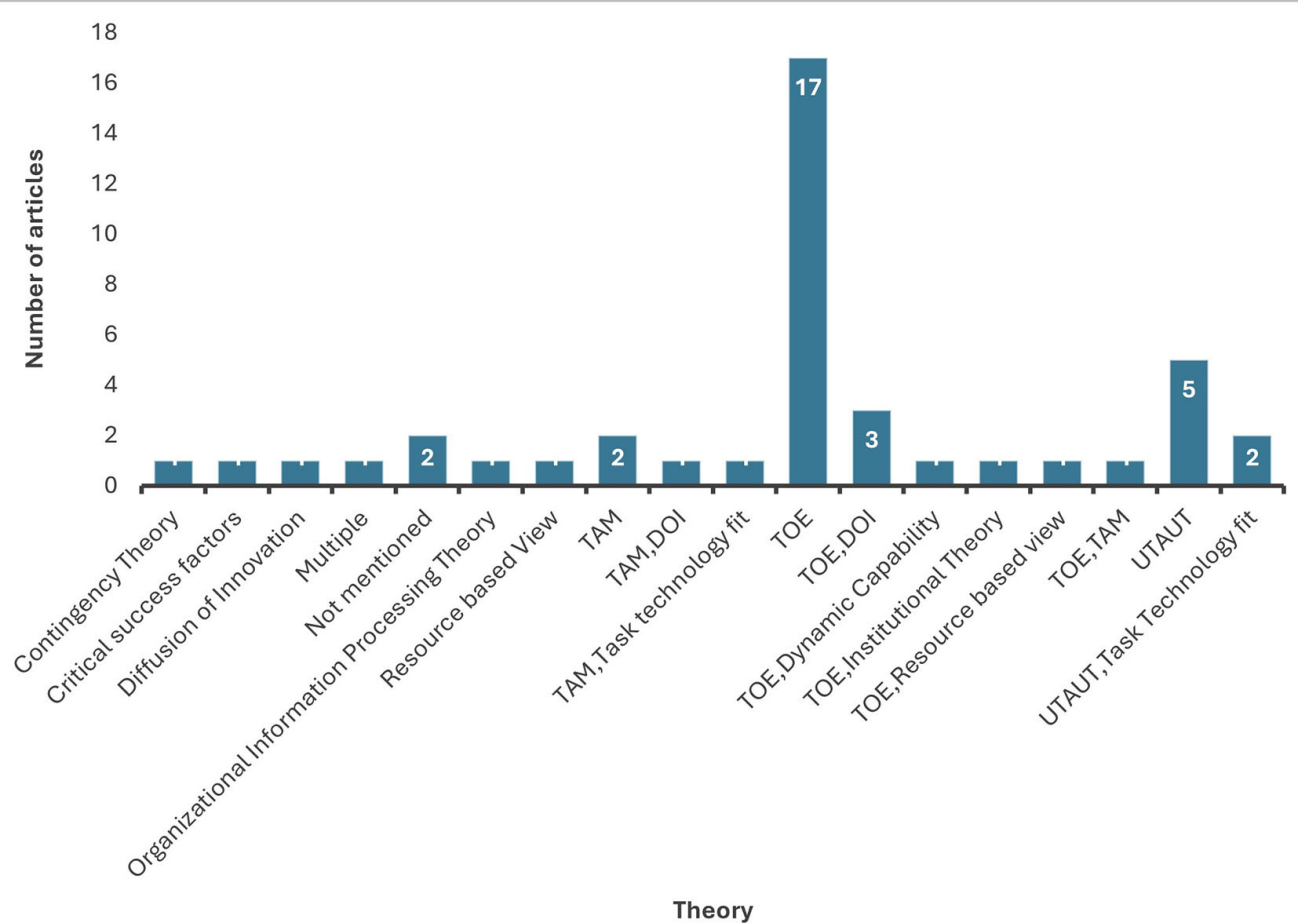
Distribution of theoretical frameworks. Source: Authors Developed, 2025. Alt Text: A bar graph representing the distribution of theoretical frameworks employed by past studies.

Figure 8 shows the different theories employed by studies considered in this review. Future researchers could integrate these theories to develop conceptual frameworks that broadly studies data analytics adoption by considering the variables thoroughly examined in the next section.

4.2.2 Key determinants of bda adoption

The current study conducted a comprehensive review of scholarly articles pertaining to the factors that influence the adoption of data analytics in both individual and organizational perspectives. To classify the key determinants of adoption, an analysis of five main dimensions, namely technological, organizational, environmental, data-related and individual factors comprising individual beliefs, personality traits, and individual capabilities, was derived from the collective findings. Tables 6- 10 explore in depth the variables in each dimension and the respective theoretical framework. Further, the tables indicate the suitability of each variable for individual or organizational level adoption. The findings will benefit future researchers to integrate two or more theoretical frameworks and thus evaluate the optimal combination suitable for data analytics adoption at firm or organizational level. Finally, the dimensions can be used to propose a comprehensive conceptual framework to help practitioners encourage data analytics adoption.

**Table 6.** Technological determinants.

Variable	Definition	Level of analysis	Theoretical framework	References
Performance Expectancy	The degree to which the individual believes that the new technology will improve their task performance	Organizational	UTAUT	A1,A3,A5,A6,A15,A33,A34
Perceived usefulness		Individual	TAM	A2,A16,A37,A41
Effort Expectancy	The ease of learning and using a new technology	Organizational	UTAUT	A1,A3,A5,A6,A15
Perceived ease of use		Individual	TAM	A2,A16,A28,A37,A41
Facilitating conditions	The extent to which the technical infrastructure available is perceived to be adequate in supporting the use of the new technology.	Organizational	UTAUT,TOE	A1,A3,A5,A6,A7,A8,A21,A22
Perceived risk	Potential for losses and uncertainties as a result of the implementation of a new technology or information system	Organizational	UTAUT, Theory of perceived risk	A1,A11,A19,A28,A29
Perceived privacy and security	The extent to which a user feels a certain system is secure and effective for transmitting and storing sensitive and/or personal information	Individual	TAM	A2,A27,A37
Compatibility	The degree to which the innovation is perceived to be consistent with the potential users’ existing values, previous experiences and requirements	Organizational	Diffusion of Innovation,TOE	A4,A7,A8,A10,A11,A14,A17,A23,A25,A26,A27,A32,A35,A36,A38,A41,A42
Complexity	The degree to which BDA technology can be regarded difficult to be understood and used for the organization	Organizational	Diffusion of Innovation,TOE	A4,A7,A8,A9,A10,A11,A17,A22,A25,A26,A27,A32,A35,A38,A40,A42
Expected benefits/Relative advantage	The degree to which BDA adoption can benefit from an organization	Organizational	Diffusion of Innovation,TOE	A4,A8,A9,A17,A19,A21,A22,A23,A24,A25,A27,A28,A31,A32,A39,A40, A41,A42
System quality	Evaluates the overall system attributes and capabilities	Individual	DeLone and Maclean IS Success model	A6
Infrastructure capabilities	Refers to reliable software applications, information systems, storage and organization networks	Organizational	TOE	A9,A28,A29,A30,A31,A39,A40
Perceived strategic value	Compares benefits with challenges	Organizational	Perceived strategic value based adoption model	A10,A14,A28,A29
Technology readiness	Defines the skills and knowledge required to leverage BDA associated applications	Organizational, Individual	TOE,Critical success factors	A10,A12,A28,A39,A42,A43
Trialability	Degree to which an innovation can be put on a trial	Organizational	TOE	A11,A27,A38
Performance and Impact Evaluation	Monitoring and evaluation of the performance of the system	Organizational	Critical success factors	A12
Observability	The degree to which the results of an innovation are visible to others	Organizational	TOE,Diffusion of Innovation	A27,A31,A38
Predictive analytics accuracy	Precision of predicting future trends by extracting information from available datasets	Organizational		A38

Source: Authors Developed, 2025.

**Table 7.** Organizational determinants.

Variable	Definition	Level of analysis	Theoretical framework	References
Facilitating conditions	The extent to which the technical infrastructure available is perceived to be adequate in supporting the use of the new technology.	Organizational	UTAUT,TOE	A1,A3,A5,A6,A7,A8, A21,A22
Top management support	The degree to which top management understands the importance of big data technology and the extent to which it is involved in related initiatives.	Organizational	TOE, Critical success factors	A3,A4,A7,A8,A9,A10,A11,A12,A14,A17,A18,A19,A22,A23,A24,A25,A26,A27,A32,A35,A36,A38,A39,A40,A41,A42,A43
Organizational readiness	Ability and willingness to make available specific organizational resources which are needed to adopt new IT innovations	Organizational	TOE	A4,A11,A14,A18,A19,A22,A23,A24,A25,A26,A27, A28,A35
Financial resources	Cost that the organization preserves to maintain new technology in the future	Organizational	TOE	A7,A8,A22,A32,A38, A40,A41
Human Expertise and skills	Refer to the employees that possess the ability and IT knowledge related to BDA	Organizational, Individual	TOE, Critical success factors	A8,A12,A13,A18,A20
Organizational resources	Refers to the tangible assets and raw materials that support programs, practice and service delivery	Organizational	TOE	A9,A38
Firm size	Size of the organization in terms of availability of more resources	Organizational	UTAUT, TOE, Institutional Theory	A10,A17,A28,A29,A38, A39,A43
Internal training	Utilizing organizations own resources and expertise to enhance skills and knowledge of others relevant to the company's needs	Individual	Contingency Theory	A20
Absorptive Capability	The capability and capacity of the firm to discover valuable information adopted from its external environment, including competitors	Organizational	TOE	A21
Supply chain connectivity	The ability of a firm to use IT to collect, analyze and disseminate information needed to synchronize decision-making across value-added activities	Organizational	TOE	A22
Information sharing	Timely, sufficient and authentic sharing of quality information within an organization	Organizational	Resource based view	A23
Nationality	Nationality of the firm/Individual	Organizational		A28
Industry Classifications	Type of industry	Organizational		A28
Organizational culture	The culture of a firm that promotes, suggestions, opinions and expressions regarding the methods and procedures. The awareness of commitment to knowledge transfer and integration within a firm	Organizational	TOE, Institutional	A38
Prior IT Experience	The firm’s experience of working with IT and related projects.	Organizational, Individual		A38,A39
Organizational Expectation	Internal pressure in organizations to make individuals comply with rules or directions	Individual	TOE	A43

Source: Authors Developed, 2025.

### 4.3 Keyword, content and co-authorship analysis

**Figure 9.**
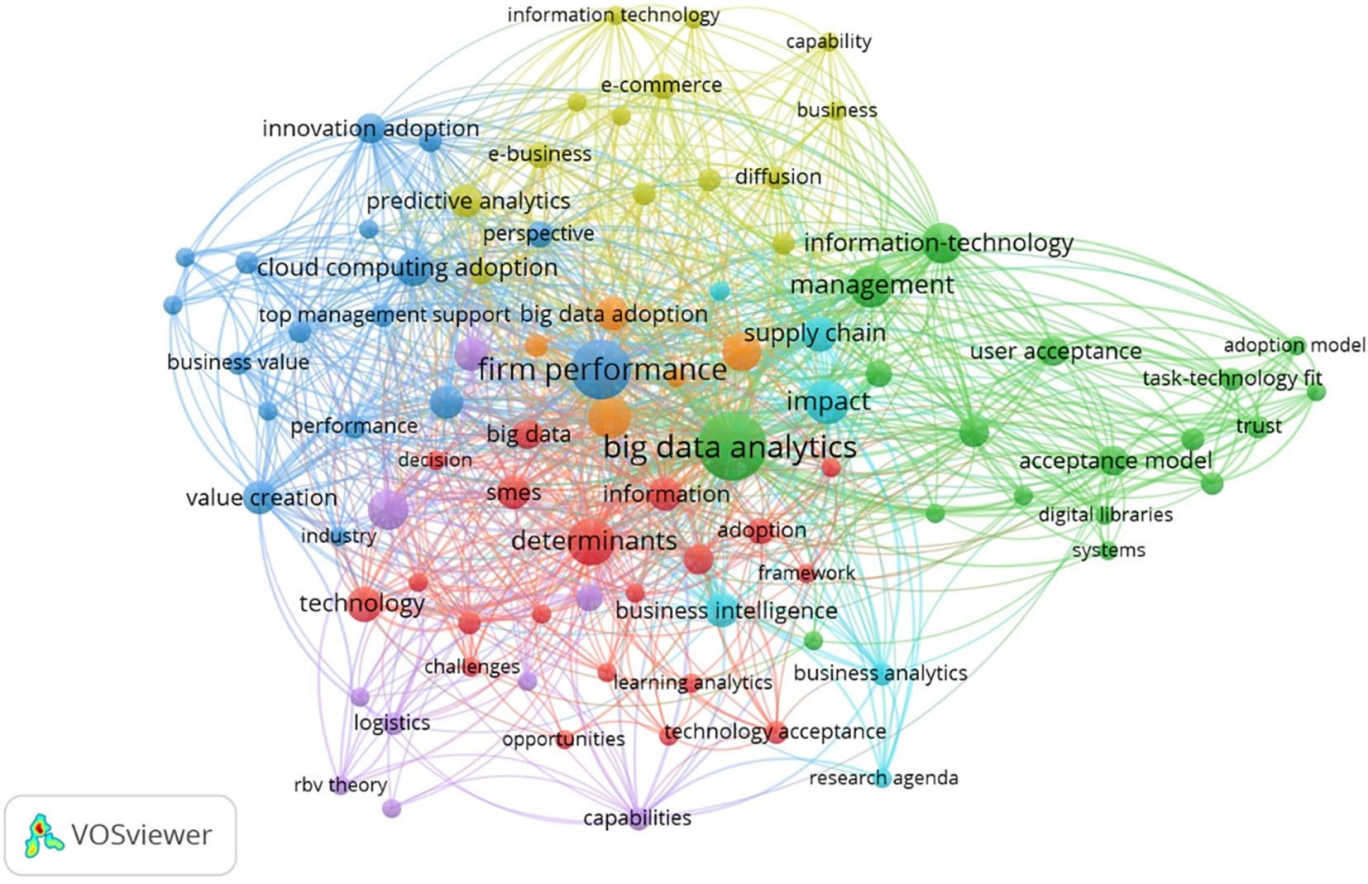
Keyword analysis. Source: VOSviewer. Alt Text: A network visualization depicting the keywords which often appear together.

To extract the most common keywords and topics of the selected paper, an analysis of the keyword occurrences was performed with VOS viewer. In particular, the analysis was aimed at the keywords used by authors, editors and publishers to link the articles published. Analysis of keywords is a process to identify and examine keywords that are important in a particular text. ^38^ Keywords were extracted from the articles selected in our analysis and subdivided into different clusters according to co-occurrence in the same work. The results of this analysis showed five main clusters setting the software with a threshold that groups together keywords that must occur at least two times and selecting only relevant keywords. The results of clusters are represented in Figure 9.

Further, Table 11 summarizes the core focus areas in BDA adoption by categorizing the findings of the keyword analysis into five main clusters. The table depicts the key themes and influential authors in the respective field. Identified research gaps are highlighted in the discussion in Section 4. The study directs future researchers to expand the key themes and combine the determinants analyzed in Tables 6-10 by integrating the theoretical frameworks that are underexplored and have less linkage in previous studies.

**Figure 10.**
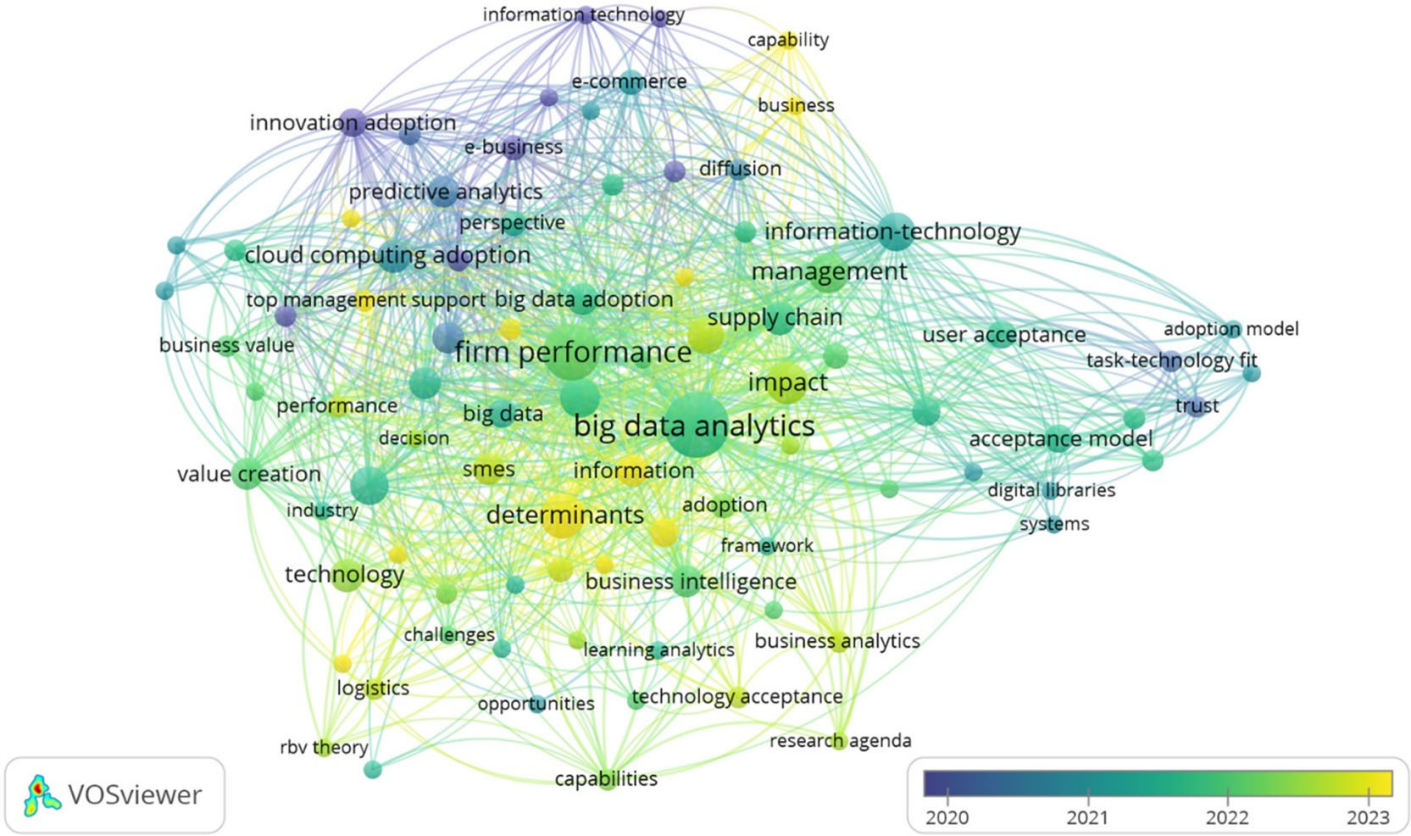
Overlay visualization diagram. Source: VOSviewer. Alt Text: A network visualization diagram where nodes represent keywords and colour of each node indicate the average publication year of the articles in which it appears. Yellow cluster represents more recent publications, whilst blue cluster represents older publications.

The analysis of Overlay Visualization diagram in Figure 10 reveals significant themes and critical gaps in the literature surrounding technology adoption, particularly concerning BDA. Central topics include the well-established connection between BDA and firm performance, with emerging trends highlighting a growing focus on value creation, organizational capabilities, and user acceptance from 2022 to 2024. However, several key research gaps persist: a weak linkage between human factors such as trust and task-technology fit with organizational outcomes; underexplored strategic enablers like top management support and Resource-Based View (RBV); and a notable lack of sector-specific studies, particularly in logistics and public institutions.

Additionally, there is a limited focus on post-adoption behaviors and value realization, highlighting a need to shift from merely understanding adoption determinants to exploring long-term benefits. The fragmented nature of theoretical frameworks indicates an opportunity for integrating models like TAM, TOE, and RBV, while emerging areas such as learning analytics require further investigation. Addressing these gaps will enhance comprehension of the multifaceted dynamics of technology adoption across varying sectors and contexts, paving the way for more comprehensive research in the future.

**Figure 11.**
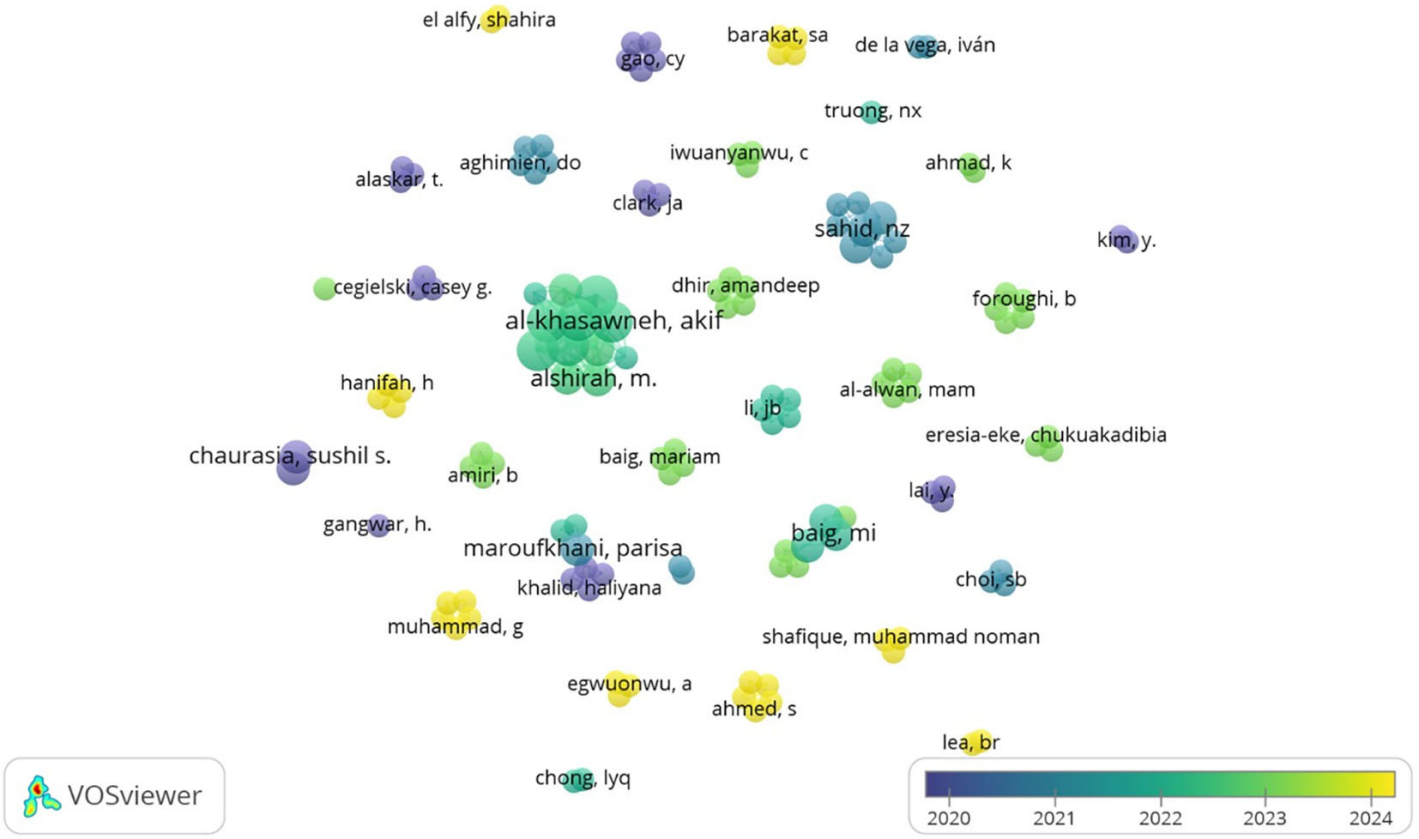
Co-Authorship analysis. Source: VOSviewer. Alt Text: A network visualization diagram analyzing the relationship between authors based on their collaborative work.

Figure 11 produced from Vos Viewer, depicts which researchers have collaborated and when they were most active. Co-authorship analysis examines the collaboration among scholars in a particular research field. ^39^ Notably, Al Khasawneh, Akif and Alshirah are central figures with broader collaborations and multiple recent researchers who have collaborated at least twice to determine antecedents of big data analytics adoption in both the retail and hospitality industry. Both studies have employed the Technology-Organizational-Environmental (TOE) framework. The clusters in yellow depict the authors who have done recent research on adopting data analytics. Authors in yellow, such as Muhammad, G., Ahmed, S., and Egwuonwu, A., are actively publishing in 2024, making them potential trendsetters or cutting-edge contributors. Recent focused topics include the adoption of learning analytics, cross-cultural studies in the adoption of BDA and the linkage between adoption and firm performance from different perspectives. Authors in blue or purple (e.g., Chaurasia, Sushil S., Cegielski, Casey G.) contributed more around 2020–2021. Their work may form the theoretical foundation or earlier findings in the field.

## 5. Discussion

This section aims to discuss the main findings, highlight research gaps and provide future research agenda and implications of theory and practices to achieve the research objectives in Section 1.

Analysis of publication trends indicates growing interest in the topic, particularly post-2018, reflecting the increasing organizational emphasis on data-driven decision making. However, the research remains unevenly distributed across industries and geographies, with a concentration in sectors like manufacturing, healthcare, finance and IT and in developed economies. ^10, 21, 31, 36, 40, 41^ There is limited research density surrounding keywords associated with specific sectors, like logistics and small and medium-sized enterprises (SMEs). This indicates a gap in sector-focused studies that could shed light on unique adoption challenges and solutions. Furthermore, the absence of substantial studies within the public sector, education, and developing countries suggests overlooked barriers and facilitators of technological adoption in these critical contexts.

A key insight from this review is that these five dimensions are deeply interconnected. Technological readiness, such as data infrastructure and analytics capabilities, often depends on organizational culture and support. Relative advantage, sometimes referred to as Perceived strategic value ^23, 42, 43^ positively influenced BD adoption. Firms which recognized the value of BDA were more inclined to adopt it than others. Similarly, individual-level factors like data literacy and user attitude are shaped by organizational training programs and leadership commitment. Environmental factors, including competitive pressure and regulatory environments, act as external motivators that influence organizational behavior. Moreover, data-related factors such as quality, availability and volume serve as foundational enablers across all other categories. The evidence suggests that determinants related to the individual are pivotal in BDA adoption. The study classifies individual factors as individual capabilities, personality traits, individual beliefs and individual behavioral factors as summarized in Table 8. Empirical investigations have indicated that adoption of emergent technologies may necessitate the incorporation of “soft skills” alongside behavioral intentions, technical proficiencies and domain-specific knowledge. ^21^ The study further adds that it is imperative to consider social influences, belief systems and contextual factors when advocating for the adoption of novel technologies. Human expertise and skills, which produced similar outcomes across all findings, was the most employed determinant and a significant factor for adoption across education, finance, manufacturing and supply chain sectors at both organizational and individual levels. ^11, 27, 40, 44, 45^

**Table 8.** Individual related determinants.

Variable	Definition	Level of analysis	Theoretical framework	References
Resistance to use	Consists of negative reactions to change or new system implementation	Organizational	UTAUT	A1,A28
Perceived privacy and security	Extent to which a user feels a certain system is secure and effective for transmitting and storing sensitive and/or personal information	Individual	TAM	A2,A27,A37
Employee readiness	Employee’s degree of expertise, attitude toward change, and perceptions of the end user’s benefits of adopting the technology.	Individual		A3
Personal Innovation	The willingness of any individual to try out any technology	Individual	Diffusion of Innovation	A3,A6
Human Expertise and skills	Refer to the employees that possess the ability and IT knowledge related to BDA	Organizational, Individual	TOE,Critical success factors	A8,A12,A13,A18,A20
Self Instruction	The extent to which educators adapt their instructional strategies according to students’ learning needs	Individual		A15
Critical thinking skills	Ability to analyze information objectively, identify biases and form well reasoned judgements	Individual	Contingency Theory	A20
Fraud detection risk responsibility	Ability to identify frauds and support risk management with analytics	Individual	Contingency Theory	A20
Fear appeal	The way of communication in a persuasive way that could change the behavior of individuals and encourage them to perceive a threat or develop a feeling of having fear	Individual	UTAUT	A30
Statistical Background	Quantitative/Analytical skills of the employee	Individual	UTAUT	A30,A43
Initial trust	A person’s desire to meet his/her needs without having previous experience or accuracy and relevant information	Individual, Organizational	Initial trust model	A33,A34,A37,A38
Task technology fit	The interplay between task characteristics and technology functionality	Individual	Task Technology fit model	A33,A34,A37

Source: Authors Developed, 2025.

**Table 9.** Environmental determinants.

Variable	Definition	Level of analysis	Theoretical framework	References
Social Influence	Measures the effect of what others think about the technology	Organizational	UTAUT	A1,A5,A6,A30
Strategic Orientation	Strategic decisions that an organization makes in order to establish a supportive infrastructure and behavior that is conducive to its ability to compete in today’s business environment	Organizational	CTUAT	A3
Competitive pressure	Perceived pressure from competitors that forces a firm to adopt new technology for the sake of maintaining competitiveness	Organizational	Institutional theory	A4,A8,A10,A11,A17,A19,A21,A22,A25,A26,A27,A32,A35,A38,A39,A42
Security and Privacy	Assurance that the client’s data will be kept safe	Organizational	TOE	A7,A8,A17,A25,A28,A29,A32,A40,A41
Government guidelines	Rules and regulations that the organization must follow when adopting new technology	Organizational	TOE	A7,A8,A9,A13,A22,A23,A24,A25,A26,A27
Regulatory support	Support given by a government authority for the adoption and assimilation of IT innovation	Organizational	TOE	A10,A11,A14,A19,A21,A32,A38,A39
Vendor support	Support offered by the vendors who offer open-source big data systems to encourage innovation adoption	Organizational	TOE	A17,A27,A35,A41
Environmental Uncertainty	The inability, at different levels, to establish the probability of future events and to predict the consequences of the decision accurately	Organizational	TOE	A19
Partner adoption	Behavior of other firms in partnership with the firm. Firms adopt big data to maintain good relations with partners	Organizational	TOE	A32,A38,A39,A42
Coercive Pressure	Condition where the focal firm is submissive to pressure from other institutional bodies, such as the government	Organizational	Institutional theory	A40
Normative Pressure	Pressure that institutions exert on companies to conform to shared decisions.	Organizational	Institutional theory	A40
Mimetic Pressure	Organizations imitating the behavior of similar firms in the industry to succeed	Organizational	Institutional theory	A40

Source: Authors Developed, 2025.

**Table 10.** Data-related factors.

Variable	Definition	Level of analysis	Theoretical framework	References
Big data quality	Adequate characterization of data, real-time view of data, right interpretation of results and determining the relevance of results, while addressing the trustworthiness of input data.	Organizational	TOE,Critical success factors	A10,A12,A22,A32,A38, A41,A43
Data Management	Process of ensuring accuracy, availability, accuracy and quality of large stores of data by the adequate allocation of data, people and resources	Organizational	TOE	A17,A30,A35,A38,A42
Data Volume	The amount of data qualifying as big data (Massive amount of data collected and generated)	Organizational	TOE	A24
Data Velocity	The speed at which the data is processed and generated	Organizational	TOE	A24
Data Variety	The diversity that exists in the type of data including structured, semi structured and unstructured	Organizational	TOE	A24

Source: Authors Developed, 2025.

**Table 11.** Content analysis.

Cluster	Keywords	Focus	Authors
Red Cluster: “Big Data Analytics and Adoption”	big data, determinants, adoption, technology, SMEs, learning analytics.	Factors influencing adoption of big data and analytics tools, particularly in small and medium enterprises.	Al-Azzam et al. (2023), Baig,Yadegaridehkordi, & Nasir (2023), Iranmanesh et al. (2023), Lutfi, Alsyouf et al. (2022), Maroufkhani et al. (2022), Truong (2022)
Blue Cluster: “Innovation Adoption and impact on firm performance”	Innovation adoption, cloud computing, predictive analytics, business value,firm performance	Adoption of innovative technologies and their impact on business value and organizational performance	Chong & Lim (2022), Egwuonwu et al. (2024), Kitcharoen (2023), Lutfi, Al-Khasawneh et al. (2022), Maroufkhani et al. (2020) Sekli & De La Vega 2021), Shafique et al. (2024), Sharma et al. (2023), Yu et al. (2022)
Yellow Cluster: “E-commerce and IT Diffusion”	e-commerce, diffusion, capability, perspective,business	How digital capabilities diffuse across businesses, specifically in online contexts	Oyewo et al. (2023), Sun et al. (2020), Verma & Chaurasia (2019), Hamed et al. (2024)
Green Cluster: “User Acceptance and Technology Fit”	Big data analytics, acceptance model, task-technology fit, user acceptance, trust, digital libraries	The integration of user-level adoption theories (e.g., TAM), trust, and fit between tasks and technologies on big data analytics	Al-Azzam et al. (2023), Azam & Ahmad (2023), Bahari et al. (2023), Muhammad et al. (2024), Sahid et al. (2021), Sani et al. (2021), Shahbaz et al. (2019)
Purple Cluster: “Technology Capabilities and Logistics”	logistics, capabilities, challenges, rbv theory	Resource-based views (RBV) on technology and their implications for logistics and organizational capability	Chong & Lim (2022), Lutfi, Al-Khasawneh et al. (2022), Lutfi et al. (2023)

Source: Authors Developed, 2025.

Despite the breadth of studies, several gaps persist, as evident from the content analysis conducted by Vos viewer. There is insufficient theoretical cohesion among clusters like the Technology Acceptance Model (TAM) and Resource-Based View. The division indicates a need for unified conceptual models that merge behavioral, organizational, and resource-based perspectives for a multi-level adoption study. There exists a notable gap in understanding how individual user behavior influences firm-level success. Most of the research focuses on initial adoption and its determinants, with post-adoption behaviors such as usage maturity, sustained benefits, and organizational learning being notably underrepresented. The long-term impacts on performance are also scarcely discussed, highlighting an area ripe for exploration. Understanding how organizations leverage technology after its adoption to achieve sustained benefits remains largely unexplored. While themes such as trust and organizational capabilities are present, their representation is scarce. This indicates a gap in the understanding of human-centric factors, such as Digital literacy, Resistance to change, Organizational culture, Leadership roles in technology adoption. More emphasis on these dimensions is essential for comprehensively understanding adoption dynamics and user engagement. Keywords associated with newer technologies like AI, blockchain, and edge computing are notably missing or underrepresented, reflecting a temporal lag in research adaptation to evolving technological trends. There is limited integration of how learning analytics contributes to organizational performance. This highlights the need to explore how data driven learning systems contribute to firm performance and innovation. There is limited research on how top management support influences employee level technology acceptance. Investigation of the moderating role of top management support on the technology adoption intention is vital. There is also a lack of cross-industry and interdisciplinary approaches that could explore the intersection of big data analytics (BDA) with themes like sustainability, governance, or ethics, indicating a need for more interconnected research paradigms.

The review offers a holistic approach that can guide both practical implementation and future research. For practitioners, findings emphasize the need for an integrated approach to analytics adoption that addresses technological capabilities, organizational support structures, employee engagement, external pressures and data maturity. For researchers, the results highlight the importance of expanding theoretical perspectives and applying more diverse methodologies to capture the complexity of adoption in real-world settings.

## 6. Conclusion

This study employed the SLR technique to explore the evolution of data analytics research and the determinants of data analytics adoption intention and implementation as available in scholarly literature. The Web of Science and Scopus databases were mainly selected for data retrieval, and several inclusion criteria were applied to select the final documents to be reviewed. Particularly, this study highlights that technology adoption depends not only on the organizational characteristics but also on the user’s individual beliefs, individual behavioral factors, personality traits and human capabilities. This study emphasizes the interplay between technological, organizational, environmental, individual and data-related factors which need to be considered when planning and implementing data analytics initiatives. The factors in the literature are derived from various theoretical backgrounds such as TOE, TAM, UTAUT, Task Technology fit and Institutional Theory. From these theories, it was found that TOE was employed the most for organizational level adoption. Researchers mainly utilized UTAUT to identify determinants affecting individual level adoption. Furthermore, studies have also integrated Institutional Theory, consisting of variables such as Coercive Pressure, Normative Pressure and Mimetic Pressure, the Initial Trust model, and the Theory of Perceived Risk with UTAUT to enhance the performance of the model in determining the intention to adopt data analytics at the individual level. Individual capabilities such as employee readiness, human expertise and skills, statistical background and critical thinking skills played important roles in data analytics adoption. Personality traits such as personal innovation and individual beliefs such as initial trust and perceived privacy and security have proven to significantly impact data analytics adoption. The study revealed that TOE was often coupled with Diffusion of Innovation and Critical Success Factors to examine antecedents of data analytics adoption at the organizational level. Technological and data related factors such as compatibility, complexity, relative advantage, infrastructure capabilities, system quality, information quality are crucial enablers for effective data analytics adoption. Organizational factors are identified as critical determinants of data analytics adoption. Significant organizational factors include facilitating conditions, organizational readiness, information sharing, internal training, absorptive capability and financial resources. In addition, factors related to the environmental dimension such as competitive pressure, mimetic pressure, government support, vendor and trading partner support, environmental uncertainty, and strategic orientation are found to be pivotal in influencing data analytics adoption.

This SLR contributes to the literature of SLR and data analytics adoption alike. First, this is among the first studies which presents the duality consisting of established theories and individual constructs of data analytics adoption and differentiates among individual and organizational level adoption. This evidence shows that studies on data analytics adoption may continue to develop in the future. Second, this study extends the method by providing a comprehensive and structured framework, highlighting trends in theoretical approaches, and uncovering gaps such as limited cross-industry research and underexplored individual factors through the content analysis. This study could be further utilized for other technological adoption as well. The insights from this review can guide organizations and individual users in making informed decisions and developing strategies to leverage data analytics for competitive advantage.

However, this study has some limitations. In terms of adoption, the study does not differentiate the actual usage and intention to use. However, based on the Theory of planned behavior, intention to use leads to actual usage but this may vary according to the context. Thus, future research may segregate the two levels of adoption exposure and relate it to the respective industry to project a clear understanding of the determinants at each level and sector. Secondly, the review is confined to empirical studies published in journal articles. The exclusion of other sources, such as conference proceedings and books, may result in the loss of potentially valuable insights. Thirdly, the study only considers empirical studies utilizing cross sectional data. Since data analytics adoption is an adoption process, future reviews can consider longitudinal studies to evaluate the effectiveness of data analytics adoption over time and identify the pivotal determinants which remain significant over time. Further this study generalizes data analytics adoption only. But, data analytics itself involves multiple branches in different domains, including big data analytics, business analytics, learning analytics, predictive analytics, prescriptive analytics, and descriptive analytics. Determinants may vary depending on the context of the type of analytics. Future research could distinguish the types of analytics to obtain more precise analysis results.

## Data Availability

No data associated with this article. Repository name: Antecedents of data analytics adoption: A systematic literature review from 2018-2024. https://doi.org/10.5281/zenodo.17181808 ^68^ This project contains the following extended data:
[Data Extraction sheet] [Prisma Flow Diagram] [Prisma_2020_Checklist] [Data Refined for SLR] [Appendix] PRISMA Checklist: https://doi.org/10.5281/zenodo.17181808 ^68^ Data are available under the terms of the Creative Commons Attribution 4.0 International license (CC-BY 4.0).
