# Supplementary material for: Antecedents of data analytics adoption: A systematic literature review from 2018-2024
Source: F1000Res. 2025 Nov 11;14:1026. Originally published 2025 Oct 2. [Version 2] doi: 10.12688/f1000research.170252.2 (PMC12640482; doi:10.12688/f1000research.170252.2)
Supplement: Peer review history

# Reviewer report: Reviewer response for version 2

**DOI:** 10.5256/f1000research.190673.r431836

**Published:** 2025-11-22

**Authors:** Tarun Kumar Vashishth

**Affiliations:** 1 IIMT University Meerut, Meerut, Uttar Pradesh, India; 2 School of Computer Science and Applications, IIMT University Meerut (Ringgold ID: 640447), Uttar Pradesh, Uttar Pradesh, India

I have reviewed the updated manuscript along with the authors’ responses, and I am pleased to confirm that the revisions sufficiently address all my previous concerns. The improvements in the abstract, introduction, methodological clarity, bibliometric analysis, conceptual model development, and expanded discussion have notably enhanced the overall rigor and coherence of the study.

 I therefore approve the revised manuscript and have no further comments.

Are the rationale for, and objectives of, the Systematic Review clearly stated?

No

Is the statistical analysis and its interpretation appropriate?

Partly

If this is a Living Systematic Review, is the ‘living’ method appropriate and is the search schedule clearly defined and justified? (‘Living Systematic Review’ or a variation of this term should be included in the title.)

Not applicable

Are sufficient details of the methods and analysis provided to allow replication by others?

Partly

Are the conclusions drawn adequately supported by the results presented in the review?

Yes

Reviewer Expertise:

NA

I confirm that I have read this submission and believe that I have an appropriate level of expertise to confirm that it is of an acceptable scientific standard.

# Reviewer report: Reviewer response for version 2

**DOI:** 10.5256/f1000research.190673.r431837

**Published:** 2025-11-20

**Authors:** Alexandra Theodoropoulou

**Affiliations:** 1 University of Patras, Patras, Greece

I have reviewed the revised version of the article and the authors’ response in detail. Most of my previous comments have been satisfactorily addressed: the positioning relative to prior systematic reviews is now clearer, the screening process and inclusion/exclusion criteria are better documented, the bibliometric analysis and figures are more transparent, and the discussion maps the findings more explicitly to the stated research questions and limitations, including publication and language bias. A few of my earlier, more optional suggestions were not implemented exactly as proposed, but the authors provide reasonable justifications and these do not materially affect my evaluation of the work. I have no further substantive comments to add at this stage.

Are the rationale for, and objectives of, the Systematic Review clearly stated?

Yes

Is the statistical analysis and its interpretation appropriate?

Partly

If this is a Living Systematic Review, is the ‘living’ method appropriate and is the search schedule clearly defined and justified? (‘Living Systematic Review’ or a variation of this term should be included in the title.)

Not applicable

Are sufficient details of the methods and analysis provided to allow replication by others?

Partly

Are the conclusions drawn adequately supported by the results presented in the review?

Partly

Reviewer Expertise:

ICT in Management, Decision-Making, Business Analytics, BDA Management & Tools

I confirm that I have read this submission and believe that I have an appropriate level of expertise to confirm that it is of an acceptable scientific standard.

# Reviewer report: Reviewer response for version 1

**DOI:** 10.5256/f1000research.187693.r423325

**Published:** 2025-10-23

**Authors:** Alexandra Theodoropoulou

**Affiliations:** 1 University of Patras, Patras, Greece

This article is a systematic review of research on big data analytics (BDA) and its drivers at both the individual and the organizational level. The authors searched major databases for studies published between 2018 and 2024 and, after screening, included 43 studies. They used PRISMA steps for the review and added a basic bibliometric analysis to show topic trends and co-occurring keywords. The review groups the main factors into clear themes and links them to well-known frameworks: individual beliefs mostly align with UTAUT/TAM, while organizational capabilities align with TOE/RBV. Common drivers include top-management support, data quality, and users’ performance and effort expectancies; outcomes are discussed but are thinner after adoption. The evidence is concentrated in a few regions and sectors. The paper ends with practical advice and calls for more cross-level work that connects individual beliefs to firm capabilities and then to performance.

 Are the rationale for, and objectives of, the Systematic Review clearly stated?

 Answer: Yes

 The rationale and four objectives are explicitly described early in the paper. A small improvement would be to phrase them as clear research questions and later map findings back to each one.

 Are sufficient details of the methods and analysis provided to allow replication by others?

 Answer: Partly

 Core elements are present (databases, time window, criteria), but key replication details are missing: whether dual screening was used, how conflicts were resolved, inter-rater reliability (e.g., Cohen’s κ).

 Is the statistical analysis and its interpretation appropriate?

 Answer: Partly

 The descriptive and bibliometric analyses are broadly suitable for this review, but interpretation would be stronger with reported co-occurrence/link metrics; also, low citations for recent year papers should be framed as a recency effect.

 Are the conclusions drawn adequately supported by the results presented in the review?

 Answer: Partly

 Many conclusions follow from the tables, yet the claim of “linking factors with frameworks” would be better supported by an explicit cross-level model and a short objectives-to-findings summary.

 If this is a Living Systematic Review, is the ‘living’ method appropriate and is the search schedule clearly defined and justified?

 Answer: Not applicable

 The manuscript does not position itself as a Living Systematic Review, and no update schedule is described.

**Suggestions**

Abstract

 The abstract would benefit from clearer, more informative structure. At present, it is difficult to see exactly what was done, to what corpus, and how the review advances knowledge. The authors could revise it to a single paragraph that briefly covers: (i) the purpose and specific gap addressed; (ii) the data and scope (databases searched, years covered, total records screened, number of studies included); (iii) the main results (key dimensions/frameworks and dominant patterns); and (iv) the implications plus 2–3 concrete research gaps. This will help readers quickly understand the contribution and its evidentiary basis.

Introduction

 The authors mention at the start of the Introduction that “…While there has been an apparent surge on data analytics research recently, ***comprehensive systematic reviews remain limited*** *.*”. This is not true. There are many systematic review papers on BDA and its application on various sectors. Some examples are:

 Therefore, the authors should change this statement accordingly, as it is factually incorrect. They could change that statement with something like “While numerous SLRs and bibliometric reviews on BDA exist across sectors, comprehensive syntheses that explicitly integrate individual- and organizational-level antecedents and map them to multiple frameworks (e.g., TOE, TAM, UTAUT, RBV) remain limited; this review aims to address that gap.”

 Furthermore, Introduction is too long. Since there is a Literature Review section that follows, it would be best to reduce the Introduction section. As purely a suggestion, it could be restructured as follows: (a) introductory paragraph (b) scope and motivation, (c) precise gap, (d) contribution statement and (e) structure of the paper.

Methods

 The authors could add a brief methods subsection that specifies four essentials: (1) whether dual screening was used; (2) how conflicts were resolved during screening/coding; (3) inter-rater reliability (e.g., reporting Cohen’s κ); and (4) explicit inclusion/exclusion rationales, with particular attention to language and publication-type restrictions. This would materially improve clarity and replicability.

 Also, the authors should add 2–3 sentences in the manuscript acknowledging language and publication-type restrictions as potential sources of bias.

Theory mapping

 The authors could carefully review Tables 6–10 to check that each factor is placed at the correct level (individual or organizational). If a factor does not clearly belong to one level, they could put it in a small “contextual/meso” group and add a short sentence explaining the choice. It would also help to include a simple legend that, for every factor, states three items in words: the factor itself, the level it belongs to (individual, organizational, or contextual), and the theory it is linked to. A brief note describing this classification scheme—how levels were decided and how unclear cases were handled—would make the tables easier to follow for all readers.

Frameworks

 The authors say the review “links factors with frameworks,” but the paper does not show a clear path that connects individual-level beliefs (UTAUT/TAM) to organizational capabilities (TOE/RBV) and then to outcomes. As a result, the promised integration across levels is not visible to the reader.

 The authors could add a simple figure with three stages: (1) individual beliefs from UTAUT/TAM, (2) organizational capabilities described by TOE/RBV, and (3) outcomes (e.g. adoption and performance). For each stage, include one or two concrete examples taken from the tables ( *for instance*, performance expectancy at the individual level; data governance capability at the organizational level; decision quality as an outcome). Then, add a short paragraph that explains, in plain words, how stronger individual beliefs can support capability building inside the firm, and how those capabilities then lead to better outcomes. Additionally, in the Discussion, two or three sentences could be added that close this loop, such as: *given the key beliefs and capabilities identified, managers should take a specific action*; future work should test this step-by-step path from beliefs, to capabilities, to outcomes. This small addition would make the stated framework link clear and testable.

Are the rationale for, and objectives of, the Systematic Review clearly stated?

Yes

Is the statistical analysis and its interpretation appropriate?

Partly

If this is a Living Systematic Review, is the ‘living’ method appropriate and is the search schedule clearly defined and justified? (‘Living Systematic Review’ or a variation of this term should be included in the title.)

Not applicable

Are sufficient details of the methods and analysis provided to allow replication by others?

Partly

Are the conclusions drawn adequately supported by the results presented in the review?

Partly

Reviewer Expertise:

ICT in Management, Decision-Making, Business Analytics, BDA Management & Tools

I confirm that I have read this submission and believe that I have an appropriate level of expertise to confirm that it is of an acceptable scientific standard, however I have significant reservations, as outlined above.

## References

[1] : Concurrence of big data analytics and healthcare: A systematic review. *International Journal of Medical Informatics* .2018;114: 10.1016/j.ijmedinf.2018.03.013 57-65 10.1016/j.ijmedinf.2018.03.013 29673604

[2] : Big data analytics as a tool for fighting pandemics: a systematic review of literature. *Journal of Ambient Intelligence and Humanized Computing* .2021;12(10) : 10.1007/s12652-020-02617-4 9163-9180 10.1007/s12652-020-02617-4 PMC759557233144892

[3] : Big data analytics in E-commerce: a systematic review and agenda for future research. *Electronic Markets* .2016;26(2) : 10.1007/s12525-016-0219-0 173-194 10.1007/s12525-016-0219-0

[4] : Big Data Analytics and Firm Performance: A Systematic Review. *Information* .2019;10(7) : 10.3390/info10070226 10.3390/info10070226

[5] : Managing supply chain resources with Big Data Analytics: a systematic review. *International Journal of Logistics Research and Applications* .2018;21(3) : 10.1080/13675567.2017.1369501 177-200 10.1080/13675567.2017.1369501

[6] : Data science and big data analytics: a systematic review of methodologies used in the supply chain and logistics research. *Annals of Operations Research* .2023; 10.1007/s10479-023-05390-7 10.1007/s10479-023-05390-7

[7] : Big Data Analytics in Weather Forecasting: A Systematic Review. *Archives of Computational Methods in Engineering* .2022;29(2) : 10.1007/s11831-021-09616-4 1247-1275 10.1007/s11831-021-09616-4

[8] : Leveraging Big Data Analytics for Understanding Consumer Behavior in Digital Marketing: A Systematic Review. *Human Behavior and Emerging Technologies* .2024;2024(1) : 10.1155/2024/3641502 10.1155/2024/3641502

[9] : A Systematic Review of Big Data Analytics for Oil and Gas Industry 4.0. *IEEE Access* .2020;8: 10.1109/ACCESS.2020.2979678 61183-61201 10.1109/ACCESS.2020.2979678

# Response

**Published:** 2025-11-01

**Authors:** Alqa Husni

**Affiliations:** Business, Informatics Institute of Technology, Colombo, Western Province, Sri Lanka

We sincerely thank the reviewers for their thoughtful and constructive comments, which have greatly enhanced the quality and clarity of this manuscript. Detailed responses to each point raised in the review comments are provided below, addressing the suggestions and concerns individually.

Abstract

 The abstract would benefit from clearer, more informative structure. At present, it is difficult to see exactly what was done, to what corpus, and how the review advances knowledge. The authors could revise it to a single paragraph that briefly covers: (i) the purpose and specific gap addressed; (ii) the data and scope (databases searched, years covered, total records screened, number of studies included); (iii) the main results (key dimensions/frameworks and dominant patterns); and (iv) the implications plus 2–3 concrete research gaps. This will help readers quickly understand the contribution and its evidentiary basis.

**Response: **Thanks for the suggestions. The abstract was structured into paragraphs has a requirement of the journal guidelines. Purpose and specific gap addressed are in the first paragraph. Databases searched , years covered and number of studies included are in the methods section. Total number of records screened have now been added under the methods section of the revised version. Implications plus 2-3 research gaps identified from this review have now beed added under the conclusion section

Introduction

 The authors mention at the start of the Introduction that “…While there has been an apparent surge on data analytics research recently, ***comprehensive systematic reviews remain limited*** *.*”. This is not true. There are many systematic review papers on BDA and its application on various sectors. Some examples are:

 Therefore, the authors should change this statement accordingly, as it is factually incorrect. They could change that statement with something like “While numerous SLRs and bibliometric reviews on BDA exist across sectors, comprehensive syntheses that explicitly integrate individual- and organizational-level antecedents and map them to multiple frameworks (e.g., TOE, TAM, UTAUT, RBV) remain limited; this review aims to address that gap.”

**Response: **Thanks for raising this. The statement has now been changed accordingly in the revised version

 Furthermore, Introduction is too long. Since there is a Literature Review section that follows, it would be best to reduce the Introduction section. As purely a suggestion, it could be restructured as follows: (a) introductory paragraph (b) scope and motivation, (c) precise gap, (d) contribution statement and (e) structure of the paper.

**Response:** We agree that the introduction section is too long , however , we couldn't reduce it as it disturbs the logical flow suggested. The first paragraph is an introduction to the domain. Second and third elaborates on the scope, motivation and precise gap. The fourth paragraph includes the contribution statement and final paragraph includes the structure of the paper.

Methods

 The authors could add a brief methods subsection that specifies four essentials: (1) whether dual screening was used; (2) how conflicts were resolved during screening/coding; (3) inter-rater reliability (e.g., reporting Cohen’s κ); and (4) explicit inclusion/exclusion rationales, with particular attention to language and publication-type restrictions. This would materially improve clarity and replicability.

**Response: **We agree that more methodoligical clarity is required. Section 3.2 and 3.3 have been revised by adding details of suggested methods. No dual screening was used , hence there were no requirements for conflicts to be resolved. Details of how bias was minimized have also been included in the revised version.

 Also, the authors should add 2–3 sentences in the manuscript acknowledging language and publication-type restrictions as potential sources of bias.

**Response: **Discussion section has been revised by adding the suggestion limitations as follows.

**"**Secondly, the review is confined to empirical studies published in formally peer reviewed journal articles. The exclusion of  gray literature, such as conference proceedings, technical reports and books, may introduce publication type bias and result in the loss of potentially valuable insights. The study was also restricted to literature published in English, introducing potential language bias, as relevant findings published in other languages may have been inadvertently excluded. "

Theory mapping

 The authors could carefully review Tables 6–10 to check that each factor is placed at the correct level (individual or organizational). If a factor does not clearly belong to one level, they could put it in a small “contextual/meso” group and add a short sentence explaining the choice. It would also help to include a simple legend that, for every factor, states three items in words: the factor itself, the level it belongs to (individual, organizational, or contextual), and the theory it is linked to. A brief note describing this classification scheme—how levels were decided and how unclear cases were handled—would make the tables easier to follow for all readers.

**Response: **Thanks for the suggestion. A brief note of how levels were decided and unclear cases were handled has now been added in Section 4.2.2.

 "The levels were derived based on whether the study involved adoption at an individual or organizational level. Each research article was manually reviewed to identify the level of adoption. For unclear cases, the respective theoretical framework employed was used to determine the level of adoption."

Frameworks

 The authors say the review “links factors with frameworks,” but the paper does not show a clear path that connects individual-level beliefs (UTAUT/TAM) to organizational capabilities (TOE/RBV) and then to outcomes. As a result, the promised integration across levels is not visible to the reader.

 The authors could add a simple figure with three stages: (1) individual beliefs from UTAUT/TAM, (2) organizational capabilities described by TOE/RBV, and (3) outcomes (e.g. adoption and performance). For each stage, include one or two concrete examples taken from the tables ( *for instance*, performance expectancy at the individual level; data governance capability at the organizational level; decision quality as an outcome). Then, add a short paragraph that explains, in plain words, how stronger individual beliefs can support capability building inside the firm, and how those capabilities then lead to better outcomes. Additionally, in the Discussion, two or three sentences could be added that close this loop, such as: *given the key beliefs and capabilities identified, managers should take a specific action*; future work should test this step-by-step path from beliefs, to capabilities, to outcomes. This small addition would make the stated framework link clear and testable

**Response: **Thankyou for this valuable suggestion. An integrative conceptual model has been proposed for testing and added as Figure 9 at the end of the results section. A short paragraph explaining this model has been included under the Discussion section. The implications suggested from this have been added as well. The integration across levels is now visible through this model

 Thanks and regards,

 Alqa

# Reviewer report: Reviewer response for version 1

**DOI:** 10.5256/f1000research.187693.r420801

**Published:** 2025-10-13

**Authors:** Tarun Kumar Vashishth

**Affiliations:** 1 IIMT University Meerut, Meerut, Uttar Pradesh, India; 2 School of Computer Science and Applications, IIMT University Meerut (Ringgold ID: 640447), Uttar Pradesh, Uttar Pradesh, India

**Summary of the Article**

 The paper presents a systematic literature review (SLR) on the antecedents of data analytics adoption across sectors, focusing on publications between 2018 and 2024. Using PRISMA guidelines, the authors selected 43 peer-reviewed articles primarily from Scopus and Web of Science. The study categorizes determinants influencing adoption into five dimensions — technological, organizational, environmental, individual, and data-related factors — and examines dominant theoretical frameworks such as TOE, TAM, UTAUT, DOI, and Institutional Theory.

 Bibliometric tools (VOSviewer and Excel) were applied to identify trends, influential authors, journals, and research clusters. The paper concludes that research is concentrated in developed Asian economies and manufacturing sectors, and that theoretical fragmentation persists, particularly between behavioral and resource-based views. It highlights gaps in cross-sectoral studies, post-adoption behaviors, and integration between organizational and individual adoption models.

**2. Evaluation and Detailed Feedback**

**A. Suitability of Title and Quality of Abstract**

- **Strengths:**
  - The title accurately represents the study’s scope and timeframe (2018–2024).
  - The abstract summarizes the purpose, methods, and findings concisely, indicating the use of PRISMA and bibliometric tools.
- **Areas for Improvement:**
  - The **abstract lacks specific quantitative findings**, such as the exact number of clusters identified or leading theoretical frameworks, which could enhance clarity.
  - It should briefly mention **key gaps or future research directions** to make the contribution more explicit.
  - Minor stylistic edits are recommended for conciseness and impact.

**Actionable suggestion:** Include key numeric outcomes (e.g., “43 studies analyzed; five major theoretical frameworks identified; TOE and UTAUT most used”) and highlight one or two major research gaps in the abstract.

**B. Adequacy of Literature Review and Proposed Methods**

- **Strengths:**
  - The literature review is comprehensive, synthesizing several previous systematic reviews and theoretical models.
  - Clear presentation of the **research objectives** and rationale for the study.
  - Appropriate use of **PRISMA methodology** and inclusion/exclusion criteria to ensure transparency and replicability.
- **Areas for Improvement:**
  1. **Depth of Critical Evaluation:**
    The review lists prior studies but often summarizes rather than critically contrasts them. For example, it could explicitly discuss how the current study improves upon past SLRs (e.g., Inamdar et al., 2021; Horani et al., 2023).
  2. **Methodological Justification:**
    While PRISMA is applied, the **data extraction and coding process** (e.g., how theoretical frameworks were classified or how inter-rater reliability was ensured) is insufficiently described.
  3. **Bibliometric Analysis:**
    The use of **VOSviewer** is appropriate, but the parameters (minimum occurrences, clustering algorithm) should be specified to support reproducibility.

**Actionable suggestions:**

- Expand Section 3 to describe the **screening and data extraction process** in more detail.
- Clarify **how biases were minimized** (e.g., through multiple reviewers or validation steps).
- Include a **table comparing this SLR’s contributions** to earlier reviews for clarity.

**C. Quality of Results Analysis and Interpretation**

- **Strengths:**
  - Results are structured logically — descriptive trends, theoretical frameworks, and determinant dimensions are clearly explained.
  - Tables 6–10 provide an excellent summary of variables and corresponding theories.
- **Areas for Improvement:**
  1. **Statistical Rigor:**
    While bibliometric results are visually presented (Figures 9–11), quantitative measures (e.g., link strength, total link count) from VOSviewer are missing. Including these would enhance analytical credibility.
  2. **Comparative Insight:**
    The discussion could better connect empirical findings (e.g., dominance of TOE framework) to implications for theory building.
  3. **Clarity of Figures:**
    Some figures lack sufficient labeling (e.g., Figures 8 and 10). Captions should specify what the axes or colors represent.

**Actionable suggestions:**

- Add **quantitative bibliometric metrics** (e.g., co-occurrence frequency, average citations per cluster).
- Enhance figures with **interpretive captions** that explain significance, not just description.
- Integrate results more directly with the study’s four research objectives.

**D. Discussion, Conclusion, and Scientific Soundness**

- **Strengths:**
  - The discussion effectively identifies research gaps, particularly regarding **fragmented theories** and **sectoral concentration**.
  - The conclusion appropriately summarizes theoretical implications and practical relevance.
- **Areas for Improvement:**
  1. **Link to Research Objectives:**
    The discussion should explicitly revisit each objective and summarize how it was met.
  2. **Novelty and Theoretical Integration:**
    The claim that the study “links factors with respective theoretical frameworks” is strong but needs clearer demonstration — e.g., proposing a **conceptual integrative model** that visually links frameworks (TOE, TAM, UTAUT, RBV).
  3. **Limitations:**
    The limitations are identified, but there is no reflection on **publication bias** or **language bias**, both of which affect systematic reviews.

**Actionable suggestions:**

- Include a **summary table mapping objectives to findings**.
- Add a **proposed conceptual model** summarizing integration of theories and determinant dimensions.
- Discuss potential **bias and limitations** (e.g., English-only studies, exclusion of gray literature).

**3. Recommendations and Points Requiring Revision**

 Category                               Assessment                                                     Key Actions Required 

**Title & Abstract                  **Partly satisfactory                               Add quantitative highlights and key research gaps in abstract

**Literature Review              **Partly satisfactory                                Deepen critical comparison; specify methodological rigor

**Methodology                     **Satisfactory but needs more detail       Clarify inclusion/exclusion process, coding reliability

**Results Analysis               **Satisfactory                                           Include bibliometric metrics; improve figure clarity

**Discussion & Conclusion  **Partly satisfactory                                Link findings explicitly to objectives; add conceptual model

**Scientific Soundness** **Conditionally acceptable with major revisions **Address missing methodological details, bias assessment, and integration model

**4. Overall Recommendation**

**Recommendation:** *Major Revision*

Are the rationale for, and objectives of, the Systematic Review clearly stated?

No

Is the statistical analysis and its interpretation appropriate?

Partly

If this is a Living Systematic Review, is the ‘living’ method appropriate and is the search schedule clearly defined and justified? (‘Living Systematic Review’ or a variation of this term should be included in the title.)

Not applicable

Are sufficient details of the methods and analysis provided to allow replication by others?

Partly

Are the conclusions drawn adequately supported by the results presented in the review?

Yes

Reviewer Expertise:

Big Data

I confirm that I have read this submission and believe that I have an appropriate level of expertise to confirm that it is of an acceptable scientific standard, however I have significant reservations, as outlined above.

# Response

**Published:** 2025-11-01

**Authors:** Alqa Husni

**Affiliations:** Business, Informatics Institute of Technology, Colombo, Western Province, Sri Lanka

We sincerely thank the reviewers for their thoughtful and constructive comments, which have greatly enhanced the quality and clarity of this manuscript. Detailed responses to each point raised in the review comments are provided below, addressing the suggestions and concerns individually.

**A. Suitability of Title and Quality of Abstract**

**Actionable suggestion:** Include key numeric outcomes (e.g., “43 studies analyzed; five major theoretical frameworks identified; TOE and UTAUT most used”) and highlight one or two major research gaps in the abstract.

**Response: **Key numeric outcomes were included in the methods and results section of the structured abstract respectively in the previous version. Revised version includes in detail the total number of screened articles and the final selection after application of inclusion/exclusion criteria. Few major research gaps identified from this review have now been added to the conclusion section of the structures abstract.

**B. Adequacy of Literature Review and Proposed Methods**

**Actionable suggestions:**

- Expand Section 3 to describe the **screening and data extraction process** in more detail.
- Clarify **how biases were minimized** (e.g., through multiple reviewers or validation steps).
- Include a **table comparing this SLR’s contributions** to earlier reviews for clarity.

**Response:** We agree with the reviewers that screening and data extraction process needs to be explained in detail. Accordingly , Section 3.2 and 3.3 have been revised to include more details of screening and data extraction process and how the biases were minimized. Table 1 in Section 2 has also been modified by adding a column which highlights the gap addressed by the current study in comparison with exisiting reviews in this domain.

**C. Quality of Results Analysis and Interpretation**

**Actionable suggestions:**

- Add **quantitative bibliometric metrics** (e.g., co-occurrence frequency, average citations per cluster).
- Enhance figures with **interpretive captions** that explain significance, not just description.
- Integrate results more directly with the study’s four research objectives.

**Response:** Section 4.3 has now been revised by adding quantitative metrics for keyword and co authorship analysis. Further Table 11 has been modified by adding total links , link strength and average citation per cluster extracted from VOS viewer.

 Figure 8 caption has been revised to indicate what each axes in the figure represents. Figure 11 caption has been slightly modified as well.

 The beginning of each subsection in the results section clearly directs the reader to which research question will be answered hence integrating the results more directly with the four research questions.

**D. Discussion, Conclusion, and Scientific Soundness**

**Actionable suggestions:**

- Include a **summary table mapping objectives to findings**.
- Add a **proposed conceptual model** summarizing integration of theories and determinant dimensions.
- Discuss potential **bias and limitations** (e.g., English-only studies, exclusion of gray literature).

**Response : **The discussion section now maps each research questions to findings in order separated by paragraphs . Potential bias and limitations have now been discussed in detail. 

 Further, a proposed conceptual model that visually links the frameworks has been added as Figure 9 at the end of the results section.
